# Intermittent hypoxia exacerbates anxiety in high-fat diet-induced diabetic mice by inhibiting TREM2-regulated IFNAR1 signaling

**DOI:** 10.1186/s12974-024-03160-1

**Published:** 2024-07-02

**Authors:** Wenyu Ni, Yun Niu, Sitong Cao, Chunsun Fan, Jian Fan, Li Zhu, Xueting Wang

**Affiliations:** 1 Present Address: Qidong People’s Hospital, Affiliated Qidong Hospital of Nantong University, Qidong Liver Cancer Institute, No.9, Seyuan Road, Chongchuan District, Nantong, Jiangsu 226000 China; 2 Institute of Special Environmental Medicine, Co-Innovation Center of Neuroregeneration, Nantong University, Nantong, China; 3 Medical Research Center Affiliated Hospital 2 of Nantong University, Nantong, China

**Keywords:** Type 2 diabetes mellitus, Obstructive sleep apnea, Anxiety, Microglia, TREM2, IFNAR1, Cognition

## Abstract

**Background:**

Type 2 diabetes mellitus (T2DM) and obstructive sleep apnea (OSA) are mutual risk factors, with both conditions inducing cognitive impairment and anxiety. However, whether OSA exacerbates cognitive impairment and anxiety in patients with T2DM remains unclear. Moreover, TREM2 upregulation has been suggested to play a protective role in attenuating microglia activation and improving synaptic function in T2DM mice. The aim of this study was to explore the regulatory mechanisms of TREM2 and the cognitive and anxiety-like behavioral changes in mice with OSA combined with T2DM.

**Methods:**

A T2DM with OSA model was developed by treating mice with a 60% kcal high-fat diet (HFD) combined with intermittent hypoxia (IH). Spatial learning memory capacity and anxiety in mice were investigated. Neuronal damage in the brain was determined by the quantity of synapses density, the number and morphology of brain microglia, and pro-inflammatory factors. For mechanism exploration, an in vitro model of T2DM combined with OSA was generated by co-treating microglia with high glucose (HG) and IH. Regulation of TREM2 on IFNAR1-STAT1 pathway was determined by RNA sequencing and qRT-PCR.

**Results:**

Our results showed that HFD mice exhibited significant cognitive dysfunction and anxiety-like behavior, accompanied by significant synaptic loss. Furthermore, significant activation of brain microglia and enhanced microglial phagocytosis of synapses were observed. Moreover, IH was found to significantly aggravate anxiety in the HFD mice. The mechanism of HG treatment may potentially involve the promotion of TREM2 upregulation, which in turn attenuates the proinflammatory microglia by inhibiting the IFNAR1-STAT1 pathway. Conversely, a significant reduction in TREM2 in IH-co-treated HFD mice and HG-treated microglia resulted in the further activation of the IFNAR1-STAT1 pathway and consequently increased proinflammatory microglial activation.

**Conclusions:**

HFD upregulated the IFNAR1-STAT1 pathway and induced proinflammatory microglia, leading to synaptic damage and causing anxiety and cognitive deficits. The upregulated TREM2 inT2DM mice brain exerted a negative regulation of the IFNAR1-STAT1 pathway. Mice with T2DM combined with OSA exacerbated anxiety via the downregulation of TREM2, causing heightened IFNAR1-STAT1 pathway activation and consequently increasing proinflammatory microglia.

**Graphical Abstract:**

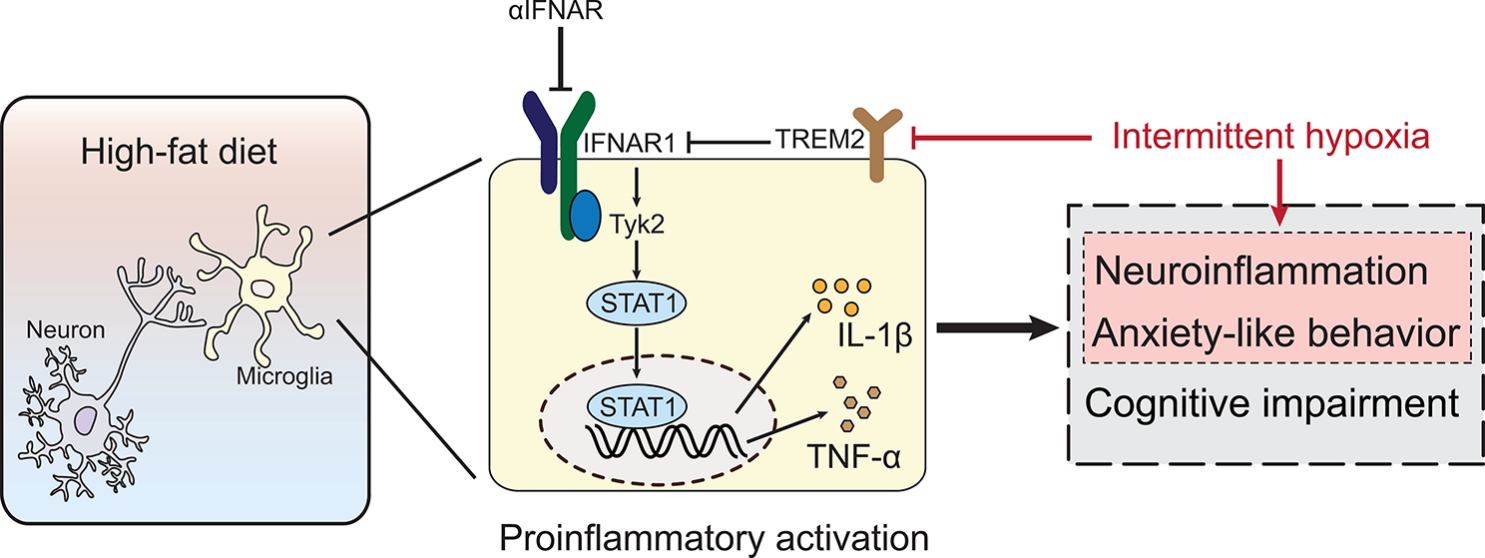

**Supplementary Information:**

The online version contains supplementary material available at 10.1186/s12974-024-03160-1.

## Introduction

Type 2 diabetes mellitus (T2DM) is a chronic metabolic disease characterized by insulin resistance, accounting for approximately 90% of all diabetic cases [1]. Cognitive dysfunction is a brain disorder potentially caused by T2DM, demonstrating characteristic structural brain abnormalities and reduced learning and memory abilities. More than 60% of people with diabetes experience mild cognitive impairment, with its prevalence increasing with age [2]. Anxiety is another brain condition induced by T2DM. People with diabetes are twice as likely to develop anxiety or depression as the general population. Moreover, people with diabetes accompanied by anxiety have a greater mental burden, more complications, a significantly lower quality of life, and a threefold higher mortality rate [3, 4]. The possible mechanisms of diabetes-associated cognitive impairment and anxiety include insulin resistance, inflammation and oxidative stress, and cerebral microvascular and lymphatic dysfunction [5–8]. However, the understanding of most individuals concerning the dangers of T2DM is limited to diabetic eye disease, diabetic nephropathy, and peripheral neuropathy, with less understanding of diabetes-induced brain disorders [9]. Therefore, a comprehensive overview of the mechanisms of diabetic encephalopathy will enhance the general awareness of the risks linked to T2DM and further improve the clinical treatment.

Obstructive sleep apnea (OSA) is one such T2DM complication as well as a high-risk factor for T2DM [10]. OSA, a sleep breathing disorder characterized by intermittent hypoxia (IH) and sleep fragmentation, has varied prevalence among patients with T2DM according to country and region, with an average incidence rate of 56.0% [11]. Both cognitive dysfunction and mood disorder are common features of OSA, which may be attributed to cerebral hypoxia, sleep fragmentation, inflammation, hormone level changes, and altered neurotransmitter concentrations. Although cognitive function impairment has been studied in the context of the comorbidity of T2DM and OSA [12, 13], the effect of T2DM complicated with OSA on anxiety and the associated mechanism remain to be elucidated.

Abnormal microglial activation is strongly associated with cognitive dysfunction and anxiety. Moreover, brain microglia have been reported to demonstrate significant activation and increased secretion of proinflammatory factors in T2DM and OSA mice models [14]. The activated microglia recognize different signals via membrane receptors and perform functions such as phagocytosis and cytokine secretion, which are involved in the neuroprotection and neurodegeneration processes [15, 16]. Additionally, the altered activation of the synaptic remodeling function in microglia causes synaptic loss, which in turn leads to cognitive impairment and anxiety [17]. The triggering receptor expressed on myeloid cells-2 (TREM2), an innate immune receptor enriched in microglial cell membranes, is a known regulator of microglia polarization and synaptic remodeling [18, 19]. TREM2 participates in clearing apoptotic cells, cellular debris, and bacteria as well as exerts anti-inflammatory effects by inhibiting secondary necrosis and the release of endogenous proinflammatory signals [20, 21]. Previous studies have indicated that TREM2 exhibits anti-inflammatory and neuroprotective effects in Alzheimer’s disease (AD) mice. TREM2 mutations promote aberrant activation of microglia in the brains of AD mice, reducing microglial reactivity to amyloid plaques, and leading to synaptic injury and cognitive impairment [22, 23]. TREM2 is also upregulated in T2DM mice, with TREM2 overexpression inhibiting microglia activation and improving synaptic transmission and ultrastructure [24]. Although TREM2 have shown neuroprotective effects in T2DM model mice, its specific mechanism is still unclear. Furthermore, the function of TREM2 in T2DM concurrent with OSA requires further exploration.

Interferons are pleiotropic cytokines with antiviral, antitumor, and immunomodulatory properties, demonstrating a crucial role in fighting viral infections [25]. Interferon-α/β receptor (IFNAR) binds to type I interferon and recruits TYK2 and JAK1 to transmit signals, resulting in the further activation of the signal transducers and activators of transcription 1 (STAT1) signaling [26, 27]. The STAT1 transcription factor is involved in regulating microglia polarization, neuroinflammation, synaptic plasticity, and cognition [28]. In this study, we initially screened the genes regulating differential expression in high-glucose (HG)-treated microglia and found significant upregulation in the IFNAR- STAT pathway, implying that TREM2 was involved in regulating the activation of HG-treated microglia by altering IFNAR signaling. Therefore, we hypothesized that the IFNAR1-STAT1 pathway might be involved in the development of cognitive impairment and anxiety in mice with T2DM combined with OSA. This study aimed to investigate the effects and the related molecular mechanism of IH-induced OSA on cognitive dysfunction and anxiety, thereby providing a theoretical basis and a new research direction for the in-depth exploration of the treatment of T2DM complicated with OSA.

## Materials and methods

### Animals and treatments

Male C57BL/6J mice (7-week-old) provided by the Experimental Animal Center of Nantong University were initially allowed to acclimate for 1 week before the study experiments. The mice were maintained at 23 ± 2 °C, 45–60% humidity, and a standard 12/12 h light/dark cycle. HFD mice were fed a 60% kcal fat diet (D12492), while normal control diet (NCD) mice were provided a 10% kcal fat diet (D12450B) from the age of 8 weeks to 33 weeks. During the administration of IH treatment, 28-week-old HFD or NCD mice were placed in a 60 cm × 30 cm × 25 cm chamber. Subsequently, rapid cycles of oxygen and nitrogen inputs were used to reduce the oxygen concentration in the chamber to 5% within 30 s, followed by maintaining this concentration for 30 s before rapidly increasing it to 20%. The next cycle was started after maintaining the oxygen concentration at 20% for 30 s. This IH exposure was performed 8 h daily for 28 days between 09:00 a.m. and 5:00 p.m. After 28 days of IH treatment, the mice underwent behavior tests and were then euthanized by anesthesia. All the study protocols involving animal experiments were reviewed and approved by the Animal Care and Use Committee of Nantong University and the Jiangsu Province Animal Care Ethics Committee (approval ID: SYXK[SU]2007-0021).

### Behavior tests

#### Open field test (OFT)

OFTs were performed to assess anxious behavior using previously reported protocols [29, 30]. Briefly, each mouse was placed in a 50 cm × 50 cm × 50 cm white polyvinyl chloride box and allowed to move freely for 5 min. An overhead camera was employed to record and analyze the movement trajectory and time spent in the peripheral (25 cm from the wall) and central zones (25 cm × 25 cm).

#### Elevated plus-maze (EPM) test

The EPM test was conducted to determine anxious behavior according to the earlier described procedure [31]. In this test, each mouse was placed in the middle of the four-arm junction of the maze (35 cm × 5 cm) and allowed to move freely for 5 min. The mice trajectories were then recorded to estimate the number of entries and time spent in the open arms.

### Novel object recognition (NOR) test

Initially, two identical objects, “A” and “B,” with a 3-cm diameter were placed in a 50 cm × 50 cm × 50 cm box at a distance of 10 cm away from the inner wall of the box. Next, individual mice were placed in the box, and their time spent exploring the objects A and B was recorded for 5 min. After 1 h or 24 h, the object B was replaced with a new object having a different appearance. The individual mice were then placed back in the box and allowed to move freely for 5 min. Finally, the exploration time of the familiar and novel object was recorded. In the NOR test, the memory capacity of the mice was obtained using a preference index calculated using the following formula: preference index (%) = TN/(TN + TF) × 100%, where TN = exploration time of novel object and TF = exploration time of familiar object. The NOR test was performed as described in prior studies [32].

### Morris water maze (MWM) test

The MWM test for investigating spatial learning-memory behavior was conducted as previously described [33]. A 150-cm circular pool was divided into four equal quadrants, i.e., northeast, southeast, southwest, and northwest quadrants. Visual cues were posted on the walls around the maze to facilitate spatial learning of the platform’s location. During the experiments, the water was made opaque with a non-toxic, white pigment and maintained at a temperature of 21 ± 1 °C. In the training period (4 times/day for 5 days), a circular platform of 10 cm in diameter was placed in the middle of the southwest quadrant at 1.5 cm below the water surface. The individual mice were released into the water and allowed to swim freely for 60 s to find the platform and stay on it for 20 s. During the probe trials (on the sixth day), the platform was removed. The individual mice were then released in the northeast quadrant and allowed to swim freely for 60 s. Video recordings were used to analyze and record the swimming trajectory, along with the estimation of the escape latency and the crossing frequency to the target quadrant.

### Cell culture and treatments

#### Primary microglia culture

Primary microglia were obtained from the cerebral cortices of 2-day-old C57BL/6J mice according to previously described protocols [34–36]. In short, brain tissue was digested with trypsin, and the resulting single-cell suspension was cultured in DMEM-F12 medium (Thermo Fisher, 11,320,033) at 37 °C in a 5% CO_2_ humidified incubator. The DMEM-F12 medium contained 10% fetal bovine serum (Celligent, CG0430B), GlutaMAX supplement (Thermo Fisher, 35,050,079), 5 ng/ml of granulocyte-macrophage colony-stimulating factor (STEMCELL Technologies, 78,017), and penicillin/streptomycin (100 U/ml and 100 mg/ml, respectively). After 10 days of growth, the mixed cell population was dominated by astrocytes and formed a fused trophoblast. Gradually, the microglia proliferated and floated in the supernatant and were harvested on the 14th day.

#### Lentivirus transfection of primary microglia

TREM2 silencing was achieved by transfecting lentivirus expressing shTrem2 (target sequence: GACCCTCTAGATGACCAAGAT) into the trophoblast cells. In this process, after 7 days of primary culture, the trophoblast cells were incubated with 8 × 10^7^ TU of lentiviruses per 25 cm^2^ culture flask and allowed to grow until microglia production. The obtained lentivirus transfection efficiency was approximately 50% in the harvested microglia.

#### Construction of shTrem2 BV2 cells

BV2 cells were transfected with shTrem2-expressing lentivirus at multiplicity of infection = 10. The positive cells were then isolated via flow cytometry at 488 nm and cultured to form single-cell clones.

#### HG treatment

Initially, cells cultured in DMEM-F12 medium (glucose concentration = 17.5 mM) were set as a control group. In the case of HG treatment, additional glucose was added to the medium to increase the final glucose concentration to 50, 75, and 100 mM. Finally, the cells were incubated in these HG mediums for 6 h to generate in vitro models of T2DM.

#### Anti-IFNAR1 antibody (αIFNAR) treatment

Cells were pretreated with 10 µg/mL of αIFNAR for 1 h, followed by co-treatment of the cells with HG for 6 h.

#### IH treatment

Cultured cells were placed in the same intermittent hypoxia chamber used for the experimental mice. In this chamber, the cells were exposed to cycles of oxygen concentration fluctuations (i.e., 20% [30 s] to 5% [30 s] of oxygen) for 6 h.

### Microglial endocytosis assay

#### Synaptosome uptake

Initially, 1 g of fresh brain tissue was well homogenized and centrifuged at 800 × *g* for 10 min, followed by aspiration of the supernatant. The supernatant was further centrifuged at 17,000 × *g* for 20 min, and the precipitate was resuspended in 0.8, 1.0, and 1.2 mol/l of sucrose gradient buffers. After centrifugation at 82,500 × *g* for 2 h, the liquid at the 1.0–1.2 mol/l sucrose interface was collected and diluted with 10 mM HEPES buffer. After centrifugation at 15,000 × *g* for 30 min, the obtained synaptosomes were resuspended in DMEM-F12 medium and then directly incubated with the microglia for 30 min. Finally, the cells were fixed and stained with synaptosome antibodies. Images were acquired using a Leica SP8 confocal microscope.

#### Dextran endocytosis

After the specified treatments, primary microglia were incubated with 100 mg/mL of 40-kDa TRITC-Dextran (AS026, ABclonal) for 30 min. The cells were then fixed and counterstained with DAPI. Images were captured utilizing a Leica SP8 confocal microscope.

### Immunofluorescence staining

Brain sections (Bregma − 1.3 to -1.9 mm) or cultured cells were fixed in 4% paraformaldehyde and subsequently permeabilized with 0.3% Triton X-100. Next, the samples were blocked in 10% donkey serum, followed by incubation with the primary antibodies at 4 °C overnight. The primary antibody binding was visualized using fluorescently labeled-secondary antibodies. Finally, the samples were counterstained with DAPI for confocal microscopy. The antibodies used for immunofluorescence staining are listed in Table 1.

**Table 1:** Antibody information

Name	Catalog number	Company
anti-IFNAR1	16-5945-85	eBioscience
anti-STAT1	66545-1-Ig	Proteintech
Anti-NeuN	MAB377	Millipore
anti-TREM2	AF1729	R&D Systems
anti-Iba1	ab5076	Abcam
anti-LAMP1	sc-20,011	Santa cruz
anti- Synaptophysin	1,101,011	Synaptic Systems
anti-PSD95	3409T	Cell Signaling Technology
anti-β-actin	66009-1-lg	Proteintech
Donkey anti-Sheep IgG Alexa 488	713-545-003	Jackson ImmunoResearch
Donkey Anti-Rat IgG Alexa 488	712-545-153	Jackson ImmunoResearch
Donkey Anti-Goat IgG Alexa 488	705-545-003	Jackson ImmunoResearch
Donkey Anti-Goat IgG Cy3	705-165-003	Jackson ImmunoResearch
Donkey Anti-Mouse IgG Cy3	715-165-150	Jackson ImmunoResearch
Donkey Anti-Rabbit IgG Cy3	711-167-003	Jackson ImmunoResearch
Donkey Anti-Rabbit IgG Alexa 647	711-605-152	Jackson ImmunoResearch
Donkey Anti-Mouse IgG Alexa 647	715-605-151	Jackson ImmunoResearch

#### Circularity coefficient analysis

The circularity coefficient analysis was performed as described before [37]. Specifically, brain tissue sections (Bregma − 1.3 to -1.9 mm) were labeled with anti-Iba1 antibodies. 10–12 Z-stacks of each mouse were captured using a 20× objective by a Leica SP8 laser scanning confocal microscope. The obtained images were maximally projected to obtain the complete morphology of the cells. Data on the number of microglia were obtained by manually counting Iba1-positive microglia in 4 pictures of each mouse. The morphological analysis of microglial cells was performed by randomly selecting 8–10 microglia/mouse and using the “shape descriptor” tool of FIJI software. The circularity coefficient is approximately close to 1 indicating increasing cell activation.

#### NeuN signal analysis

Bain tissue sections (Bregma − 1.3 to -1.9 mm) were labeled with anti-NeuN antibody. 10–12 Z-stacks of each mouse was captured using a 20X objective, zoom in 0.75-fold, by a Leica SP8 laser scanning confocal microscope. The obtained images were maximally projected. Data on the number of neurons were obtained by manually counting NeuN signal in 4 pictures of each mouse by FIJI software.

#### PSD95 and nuclear STAT1 signal analysis

Brain tissue sections (Bregma − 1.3 to -1.9 mm) were labeled with anti-Iba1 or anti-STAT1 (anti-PSD95) antibodies. 10 μm Z-stacks (0.8 μm of each stack) of each mouse were captured using a 100× objective, zoom in 2-fold by a Leica SP8 laser scanning confocal microscope. The obtained images were maximally projected to obtain the complete morphology of microglia. STAT1 intensity in nucleus of Iba1^+^ cells or PSD95 intensity in Iba1^+^ cells were counted by FIJI software. The average intensity of 10–20 microglia in each mouse was calculated as a data point.

#### Synapses number analysis

Brain tissue sections (Bregma − 1.3 to -1.9 mm) were labeled with anti-Synaptophysin and anti-PSD95 antibodies. 2 μm Z-stacks (0.2 μm of each stack) of each mouse were captured using a 100X objective, zoom in 2-fold by a Leica SP8 laser scanning confocal microscope. The obtained images were maximally projected. Signals from Synaptophysin and PSD95 within 200 nm are considered as intact synapses [38]. The total number of intact synapses was counted in 4 fields of view per mouse.

### Nissl staining

Nissl staining for counting the neurons in the hippocampal CA1 region was performed as described previously [39]. Briefly, brain Sect. (40-mm thick) were soaked in trichloromethane, followed by staining using 1% tar violet. The stained samples were then dehydrated using an ethanol gradient and xylene transparent. Images were obtained with a Leica DM4000B microscope.

### RNA-seq and qRT-PCR

Initially, purified total RNA was isolated from the brain tissue or cells using TRIzol buffer. The purified total RNA was then submitted to OBiO Technology for RNA-seq. Differentially Expressed Genes (DEGs) were identified using an empirical Bayes linear model, and a P-value was calculated for the expression of all genes. A threshold of P-value < 0.05 and fold-change ≥ 2 was established. The DEGs were visualized and clustered in a heatmap using the pheatmap package (version 1.0.12, http://cran.r-project.org/web/packages/pheatmap) in R. The clusterProfiler tool (http://bioconductor.org/packages/release/bioc/html/clusterProfiler.html) was utilized to carry out Kyoto Encyclopedia of Genes and Genomes (KEGG) analyses for the differentially expressed genes that were up- and down-regulated [40]. The criteria for significance were set at a P-value < 0.1, adjusted using the Benjamini and Hochberg method.

First-strand cDNA was synthesized utilizing the HiScript III 1st Strand cDNA Synthesis Kit (Vazyme Biotech, R312-02). Ultimately, gene expression was measured via qRT-PCR using the AceQ qPCR SYBR Green Master Mix (Vazyme Biotech, Q141-02), which was performed at a cycle setting of 95 °C for 10 s, 60 °C for 30 s, and 72 °C for 20 s for 40 cycles. The relative amount of gene expression was calculated using ΔCt values. The primers employed for qRT-PCR are presented in Table 2.

**Table 2:** Primer information

Name	Direction	Sequence (5′—3′)	
*Cd86*	Forward	ACGTATTGGAAGGAGATTACAGCT	
	Reverse	TCTGTCAGCGTTACTATCCCGC	
*Fcgr3*	Forward	TTTGGACACCCAGATGTTTCAG	
	Reverse	GTCTTCCTTGAGCACCTGGATC	
*Arg1*	Forward	CATTGGCTTGCGAGACGTAGAC	
	Reverse	GCTGAAGGTCTCTTCCATCACC	
*Mrc1*	Forward	GTTCACCTGGAGTGATGGTTCTC	
	Reverse	AGGACATGCCAGGGTCACCTTT	
*Il1b*	Forward	TGCCACCTTTTGACAGTGATG	
	Reverse	TGATGTGCTGCTGCGAGATT	
*Tnfa*	Forward	AAGCCTGTAGCCCACGTCGTA	
	Reverse	GGCACCACTAGTTGGTTGTCTTTG	
*Tgfb*	Forward	TGATACGCCTGAGTGGCTGTCT	
	Reverse	CACAAGAGCAGTGAGCGCTGAA	
*Trem2*	Forward	ATGACACCCTTGCTGGAACC	
	Reverse	GCTAGAGGTGACCCACAGGA	
*Ifnar1*	Forward	CCAAGGCAAGAGCTATGTCCTG	
	Reverse	CAGTGCGTAGTCTGGACATTTGC	
*Tyk2*	Forward	GCTTTCCTGCATGGTGTTTGCG	
	Reverse	TGTCGCCGTAACCACACATCCA	
*Stat1*	Forward	TACGGAAAAGCAAGCGTAATCT	
	Reverse	TGCACATGACTTGATCCTTCAC	
*Actb*	Forward	CATCCGTAAAGACCTCTATGCCAAC	
	Reverse	ATGGAGCCACCGATCCACA	
*Trem2-OE*	Forward	ACAGCACCTCCAGGAATCAAG	
	Reverse	AGGATCTGAAGTTGGTGCCC	

### Protein isolation and western blot

Brain tissue or cells were lysed in RIPA buffer, and the protein concentration was estimated using the bicinchoninic acid assay. Proteins were then isolated from the remaining lysate and transferred to polyvinylidene fluoride membranes for hybridization with primary antibodies. Primary antibody binding was detected by HRP-conjugated secondary antibodies. The antibodies used for Western blot are provided in Table 1.

### Statistical analysis

FIJI software (National Institutes of Health) was employed to calculate the fluorescent signal intensities on the microscopy images and the grayscale values of the Western blot protein bands. All data were analyzed via GraphPad Prism version 8.0 software (GraphpPad). The Student’s *t*-test and two-way ANOVA followed by Dunnett’s multiple comparisons procedures were applied for statistical assessment. Data were presented as mean ± Standard Error of the Mean. The significance levels for all graphs in this study were as follows: *p < 0.05, **p < 0.01, and ***p < 0.001. n.s. indicated no statistical difference.

## Results

### HFD-induced diabetic mice exhibit anxiety-like behavior and cognitive impairment

**Fig. 1:**
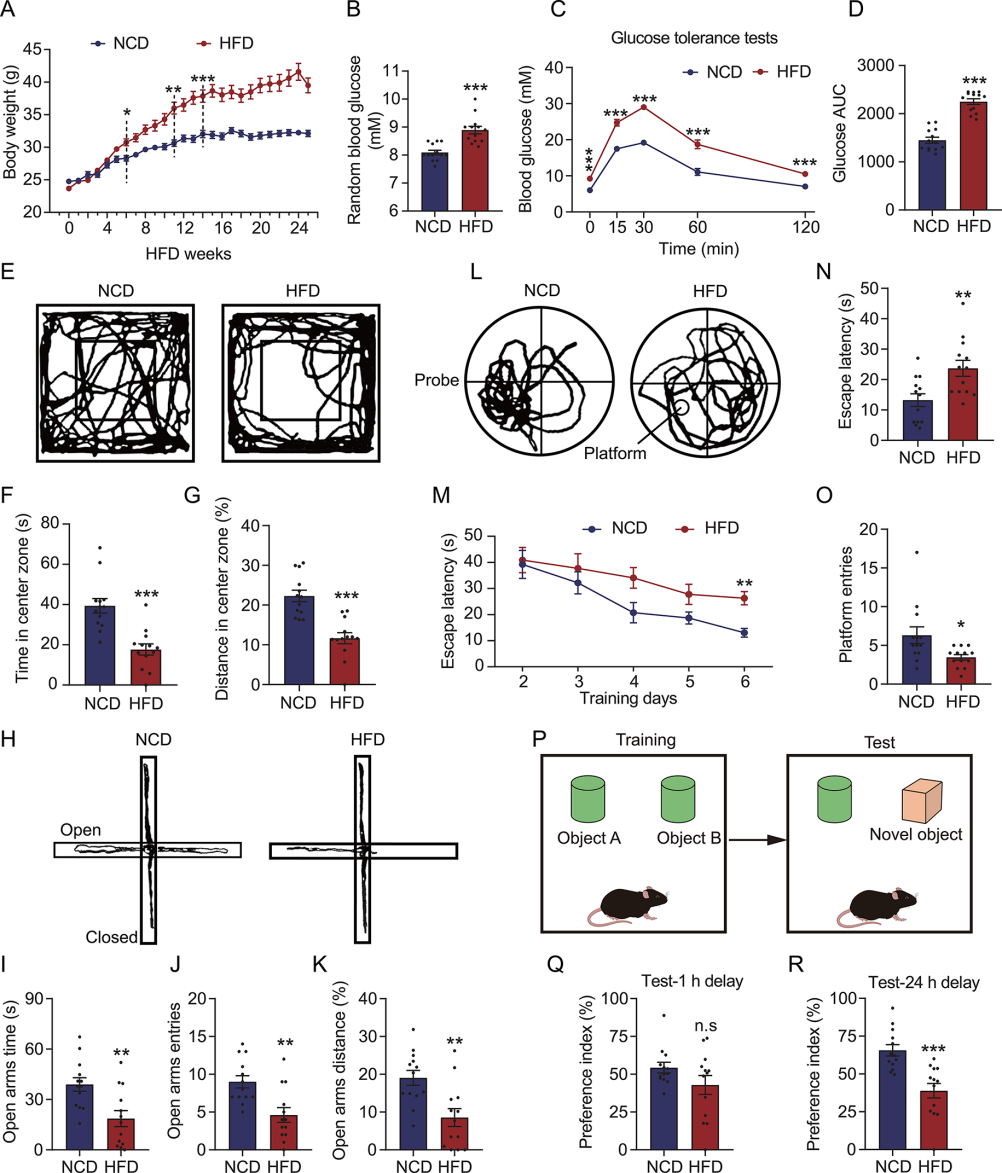
HFD mice exhibit elevated blood glucose, cognitive impairment, and anxiety. (**A**) Weekly body weight of the HFD mice. (**B**) Random blood glucose concentration of the mice after 26 weeks of HFD. (**C**) Glucose tolerance test findings of the mice after 26 weeks of HFD. (**D**) Area under the curve (AUC) for the glucose tolerance test results in panel **C**. (**E**) Movement trajectory of the mice in the OFT. (**F**) Dwell times of the mice in the central zone during the OFT. (**G**) Percentage of the distance traveled by the mice in the central zone of the open field. (**H**) Movement trajectory of the mice in the EPM test. (**I**) Time spent by the mice in the open arm of the maze. (**J**) Frequency of mice entries in the open arm of the maze. (**K**) Percentage of distance traveled by the mice in the open arm of the maze. (**L**) Swimming trajectory of the mice during the probe period of the MWM test. (**M**) Escape latency (i.e., the time to reach the target platform) of the mice during the training period of the MWM test. (**N**) Escape latency of the mice to reach the target for the first time during the probe period of the MWM test. (**O**) Frequency of crossing the target platform area during the probe period of the MWM test. (**P**) Schematic diagram of the NOR experiment. During the training stage, the mice are allowed to move freely in a box and familiarize themselves with two identical objects. After 1–24 h, one familiar object is replaced with a novel object, and the frequency of explorations of the familiar and novel objects in 5 min is measured. (**Q**) Preference index of the mice for the novel object in the NOR test after a 1-h delay. (**R**) Preference index of the mice for the novel object in the NOR test after a 24-h delay. *n* = 13. *p < 0.05, **p < 0.01 and ***p < 0.001 by Student’s *t*-test. n.s. indicates no significant difference. HFD, high-fat diet; OFT, open field test; EPM, elevated plus maze; MWM, Morris water maze

We induced T2DM in mice using a 60% kcal HFD to investigate whether cognitive impairment may develop in T2DM mice. As shown in Fig. 1A and s1A, the HFD mice showed significant weight gain and reduced appetite. Furthermore, the mice exhibited higher fasting glucose (Fig. s1B) and random glucose levels (Fig. 1B) as well as poorer glucose tolerance compared to the NCD mice (Fig. 1C and D), establishing that the HFD successfully induced T2DM in the mice. Subsequently, we examined the anxiety-like behaviors of the HFD mice using OFT and EMP test. In the OFT, the mice demonstrated reduced entries (Fig. 1E), dwell times (Fig. 1F), and the ratio of distance traveled (Fig. 1G) in the central zone of the field. Consistently, the EPM test results showed that HFD mice had decreased exploration (Fig. 1H) as well as lower time spent (Fig. 1I), entries (Fig. 1J), and the ratio of movement distance (Fig. 1K) in the open arm. Neither distance traveled nor average speed of mice during the tests of mice changed in the OFT and EPM tests (Fig. s2). All these results suggested that HFD induces anxiety-like behavior in HFD-induced diabetic mice. We also explored the effects of HFD on the spatial learning-memory capacity of the mice via the MWM and NOR experiments. As depicted in Fig. 1L and M, the HFD mice exhibited reduced exploration of the target quadrant and significantly poorer learning ability in the water maze than the NCD mice. In the probe trials, the escape latency (i.e., the time to locate the target platform quadrant) of the HFD mice was prolonged (Fig. 1N), along with significantly reduced entries in the target quadrant (Fig. 1O). In the 1 h-delay test of the NOR experiment, the HFD mice did not significantly differ from the NCD mice in the preference index (Fig. 1Q). However, the HFD mice showed a significantly lower preference index than the NCD mice in the 24-h delay test (Fig. 1R), indicating a loss of prolonged memory in the HFD mice. Considering these findings, HFD-induced T2DM mice may exhibit prominent anxiety-like behavior and cognitive impairment.

**Fig. 2:**
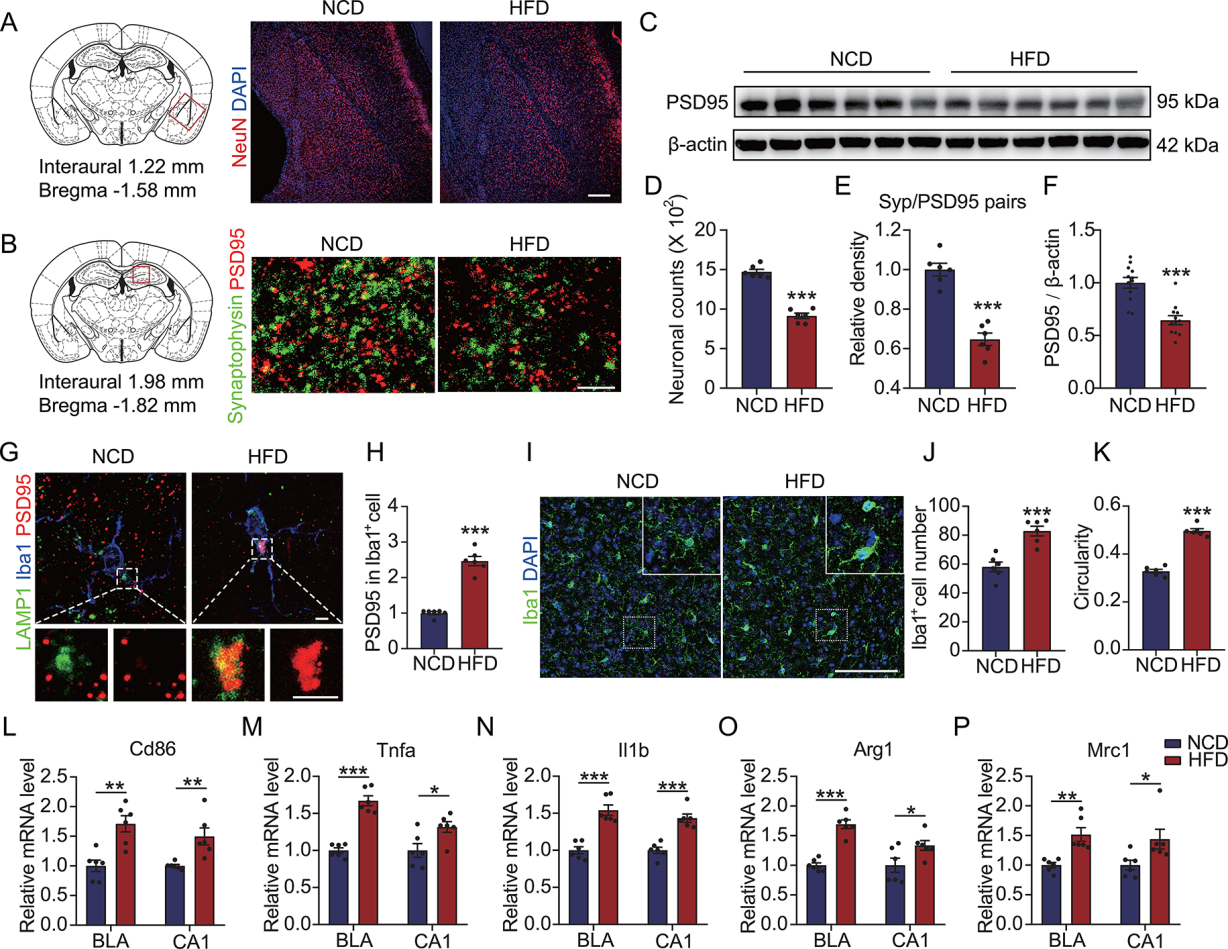
Upregulation of synaptic phagocytosis and proinflammatory polarization of microglia occur in HFD mice brains. (**A**) Brain sections of the HFD mice were probed with anti-NeuN antibody and counterstained with DAPI to obtain microscopy images of the basolateral amygdalar complex (BLA) region. Scale bar = 200 μm. (**B**) Brain sections of the HFD mice were labeled with anti-PSD95 and anti-synaptophysin antibodies to acquire microscopy images of the hippocampal CA1 region. Scale bar = 2 μm. (**C**) PSD95 in the lysate of mouse brain tissue was detected via Western blot. (**D**) The number of neurons in the BLA region in panel **A.** (**E**) Colocalization ratio of PSD95 and synaptophysin in panel **B**. (**F**) Grayscale values of the bands in panel **C** (*n* = 12). (**G**) Brain sections of the HFD mice were labeled with anti-LAMP1, anti-PSD95, and anti-Iba1 antibodies to obtain microscopy images of the microglia in the BLA region. Scale bar = 3 μm. (**H**) PSD95 intensity in the Iba1^+^ cells in panel **G**. (**I**) Brain sections of the HFD mice were labeled with anti-Iba1 antibodies and counterstained with DAPI to capture microscopy images of the BLA region. Scale bar = 100 μm. (**J**) The number of Iba1^+^ cells in panel **I**. (**K**) Circularity coefficients of the Iba1^+^ cells in panel **I** were calculated using FIJI software. (**L** to **P**) Expressions of *Cd86*, *Tnfa*, *Il1b*, *Arg1*, and *Mrc1* in the BLA and CA1 regions were measured via qRT-PCR. *n* = 6, *p < 0.05, **p < 0.01, and ***p < 0.001 by Student’s *t*-test. HFD, high-fat diet; BLA, basolateral amygdalar

### HFD induces microglial activation and synaptic phagocytosis to trigger neuronal damage in diabetic mice brains

Next, we investigated the morphology of the anxiety-related basolateral amygdalar complex (BLA) region [41] and the learning memory-related hippocampal CA1 region [42]. Our results showed that the number of neurons was significantly reduced in the BLA region of the HFD mice (Fig. 2A and D). Although no significant reduction of neurons was observed in the dentate gyrus (DG) and CA1 region (Fig. s3A to C), the synapses were significantly decreased in the HFD mice brains (Fig. 2B and E, Fig. s3E to H). In line with this finding, reduced intensity of total brain PSD95 was detected in the HFD mice (Fig. 2C and F), demonstrating an aberrant synaptic plasticity in the HFD mice brains. Given the regulatory role of microglia in synaptic plasticity [43], we subsequently examined synaptic uptake by the microglia in the HFD mice brains. Our results revealed significantly increased colocalization of PSD95 with LAMP1 in the Iba1^+^ cells in the BLA (Fig. 2G and H) and CA1 regions (Fig. s3I and J), indicating that the reduction in synapses might be related to the enhanced synaptic uptake by the microglia in the HFD mice brains. In support of this hypothesis, we further observed microglial activation in the BLA (Fig. 2I to K) and CA1 regions (Fig. s3K to M) in the HFD mice. Moreover, we verified that the polarization markers *Cd86*, *Arg1*, and *Mrc1* were significantly upregulated and that the proinflammatory factors TNF-α and IL-1β were significantly elevated in the microglia of the HFD mice brains (Fig. 2L to P). All these results suggested that HFD induces cognitive impairment by promoting the proinflammatory activation of microglia, which in turn enhances the aberrant uptake of synapses.

**Fig. 3:**
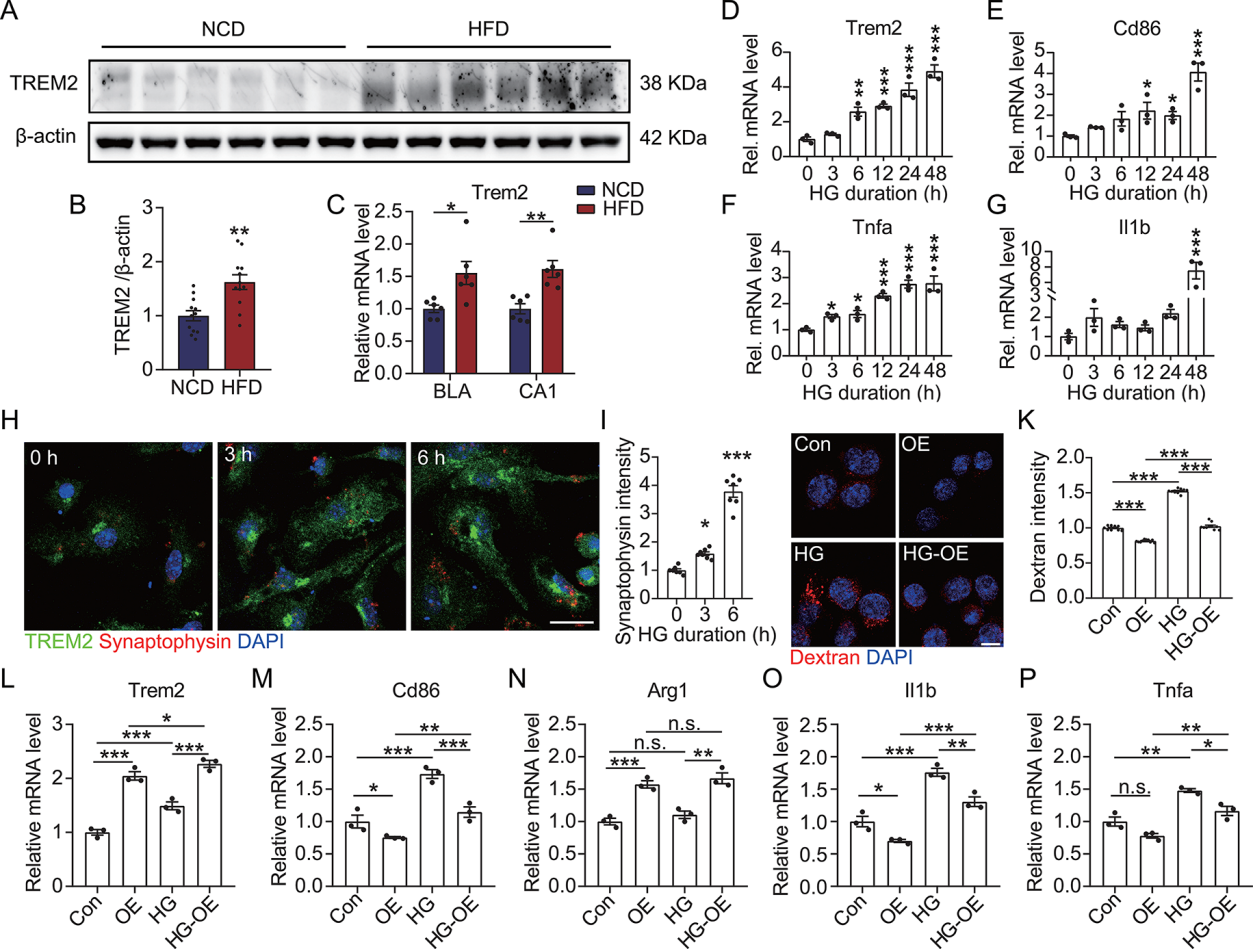
Upregulation of TREM2 inhibits HG-induced proinflammatory polarization of microglia. (**A**) TREM2 levels in brain tissue lysate were detected using Western blot analysis. (**B**) Grayscale values of the TREM2 bands in panel **A** (*n* = 12). (**C**) *Trem2* expression in the BLA and CA1 regions was determined using qRT-PCR (*n* = 6). (**D** to **G**) Expressions of *Trem2*, *Cd86*, *Tnfa*, and *Il1b* were estimated via qRT-PCR in the primary microglia treated with 50 mM of HG for specified durations (*n* = 3). (**H**) After HG treatment for the indicated durations, primary microglia were incubated with synaptosomes for 30 min. The cells were then probed with anti-TREM2 and anti-synaptophysin antibodies and counterstained with DAPI. Scale bar = 20 μm. (**I**) Synaptophysin intensity in the TREM2^+^ cells in panel **H** (*n =* 7). (**J**) After HG treatment for 6 h, TREM2-overexpressing (OE) BV2 cells were incubated with 40-kDa TRITC-Dextran for 30 min, followed by fixation and counterstaining with DAPI. Scale bar = 10 μm. (**K**) TRITC intensity per cell in panel **J** (*n =* 10). (**L** to **P**) Expressions of *Trem2*, *Cd86*, *Arg1*, *Il1b*, and *Tnfa* were measured by qRT-PCR in TREM2-OE BV2 cells after HG treatment (*n* = 3). *p < 0.05, **p < 0.01, and ***p < 0.001 by one-way ANOVA (**D** to **G**, **I**, and **K**) or two-way ANOVA (**L** to **P**). n.s. indicates no significant difference

### Upregulated TREM2 inhibits the proinflammatory polarization of HG-treated microglia

TREM2 is a membrane receptor primarily expressed on microglia in the central nervous system, exhibiting crucial involvement in synaptic protection, regulating microglial phenotypic transformation, and anti-inflammatory processes [20, 21]. Similar to a previous study that demonstrated TREM2 upregulation in the brains of T2DM mice [24], this study found heightened TREM2 levels in the HFD mice brains (Fig. 3A to C). Furthermore, we treated primary microglia with different glucose concentrations to investigate whether the HG environment was responsible for TREM2 upregulation and proinflammatory microglial activation. Our results showed that administrating 50 mM of glucose (HG treatment) caused a significant upregulation of TREM2 and the proinflammatory markers CD86, TNF-α, and IL-1β in the microglia (Fig. s4). However, this upregulation did not exhibit a significant concentration dependence. Subsequently, we treated the microglial cells with HG for different durations, revealing that the HG-induced upregulation of TREM2 and the proinflammatory markers was time-dependent (Fig. 3D to G). Correspondingly, we observed that HG activated the microglial uptake of synapses (Fig. 3H and I), suggesting that the HG extracellular environment contributed to the activation and synaptic phagocytosis activity of the microglia.

**Fig. 4:**
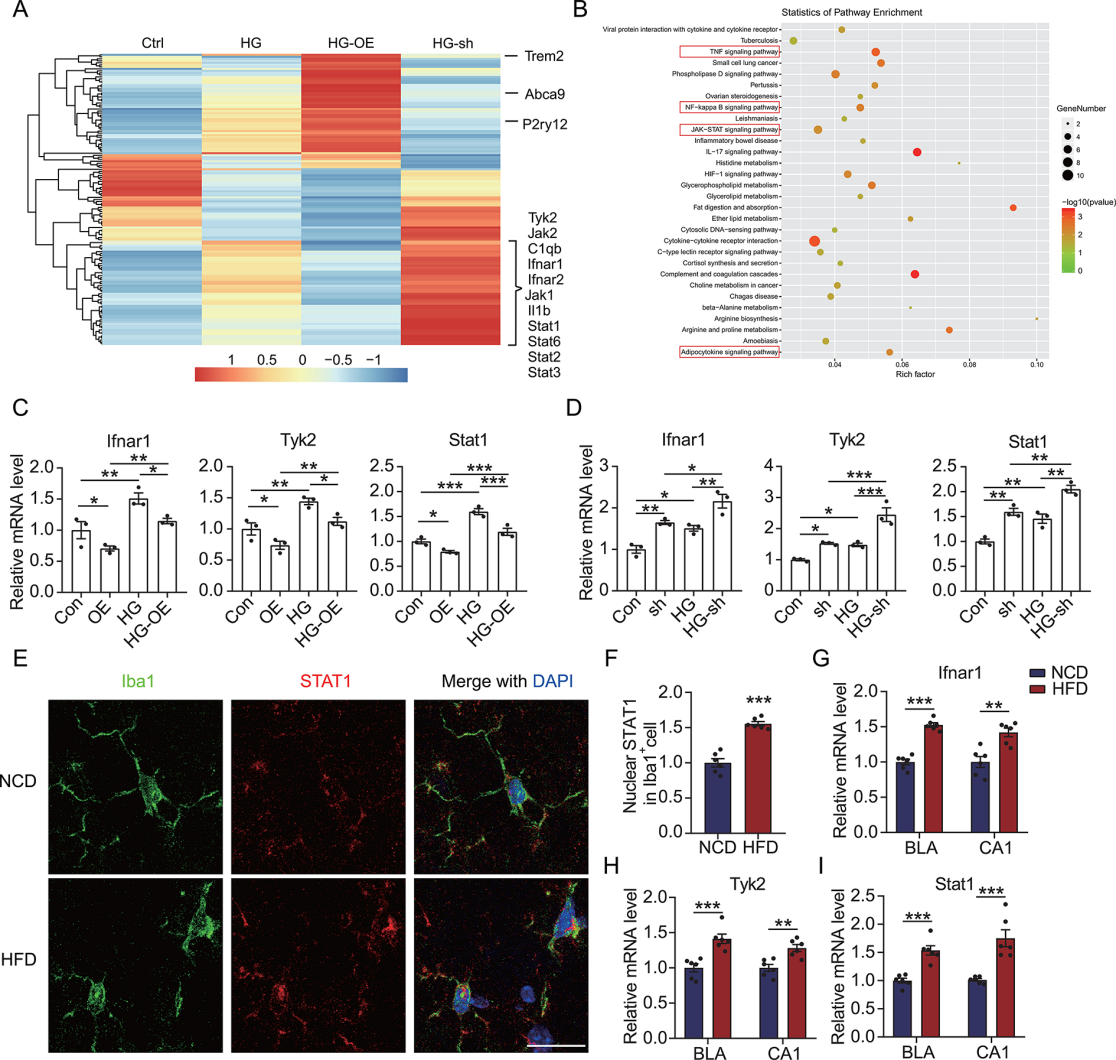
TREM2 negatively regulates IFNAR1-STAT1 signaling in HG-treated microglia. (**A** to **D**) Total RNA of HG-treated TREM2-OE or shTrem2 BV2 cells was isolated for RNA-seq (*n* = 3). (**A**) A heatmap demonstrating the differentially expressed genes. (**B**) KEGG pathway analysis depicting the enriched pathways. (**C** and **D**) Expressions of *Ifnar1*, *Tyk2*, and *Stat1* in the HG-treated TREM2-OE or shTrem2 BV2 cells were detected using qRT-PCR. (**E**) Brain sections of the HFD mice were labeled with anti-Iba1 and anti-STAT1 antibodies and counterstained with DAPI, followed by microscopy imaging of the microglia in the BLA region. Scale bar = 20 μm. (**F**) Nuclear STAT1 intensity in the Iba1^+^ cells in panel **E** (*n* = 6). (**G** to **I**) Expressions of *Ifnar1*, *Tyk2*, and Stat1 in the BLA and CA1 regions of the HFD mice were detected using qRT-PCR (*n* = 6). *p < 0.05, **p < 0.01, and ***p < 0.001 by two-way ANOVA (**C** and **D**) or Student’s *t*-test (**F** to **I**)

Based on the anti-inflammatory functions of TREM2, we further verified the regulatory role of TREM2 in HG-treated microglia. Our findings indicated that upregulated TREM2 (Fig. 3L) significantly restored HG-induced excessive phagocytosis in the microglia (Fig. 3J and K), leading to a significant attenuation of the microglia polarization to the proinflammatory type (Fig. 3M to P). Moreover, microglial synaptic phagocytosis was significantly upregulated after the silencing of TREM2 (Fig. s5A), with further upregulation detected following TREM2 silencing in the HG-treated microglia (Fig. s5B and C). Lastly, silencing TREM2 also exacerbated the HG-induced proinflammatory activation of the microglia (Fig. s5D to G). All these results demonstrated that upregulated TREM2 inhibited the further enhancement of proinflammatory activation and synaptic phagocytosis activity of the HG-treated microglia. Therefore, TREM2 upregulation under HG conditions may be a mechanism of cellular compensation.

**Fig. 5:**
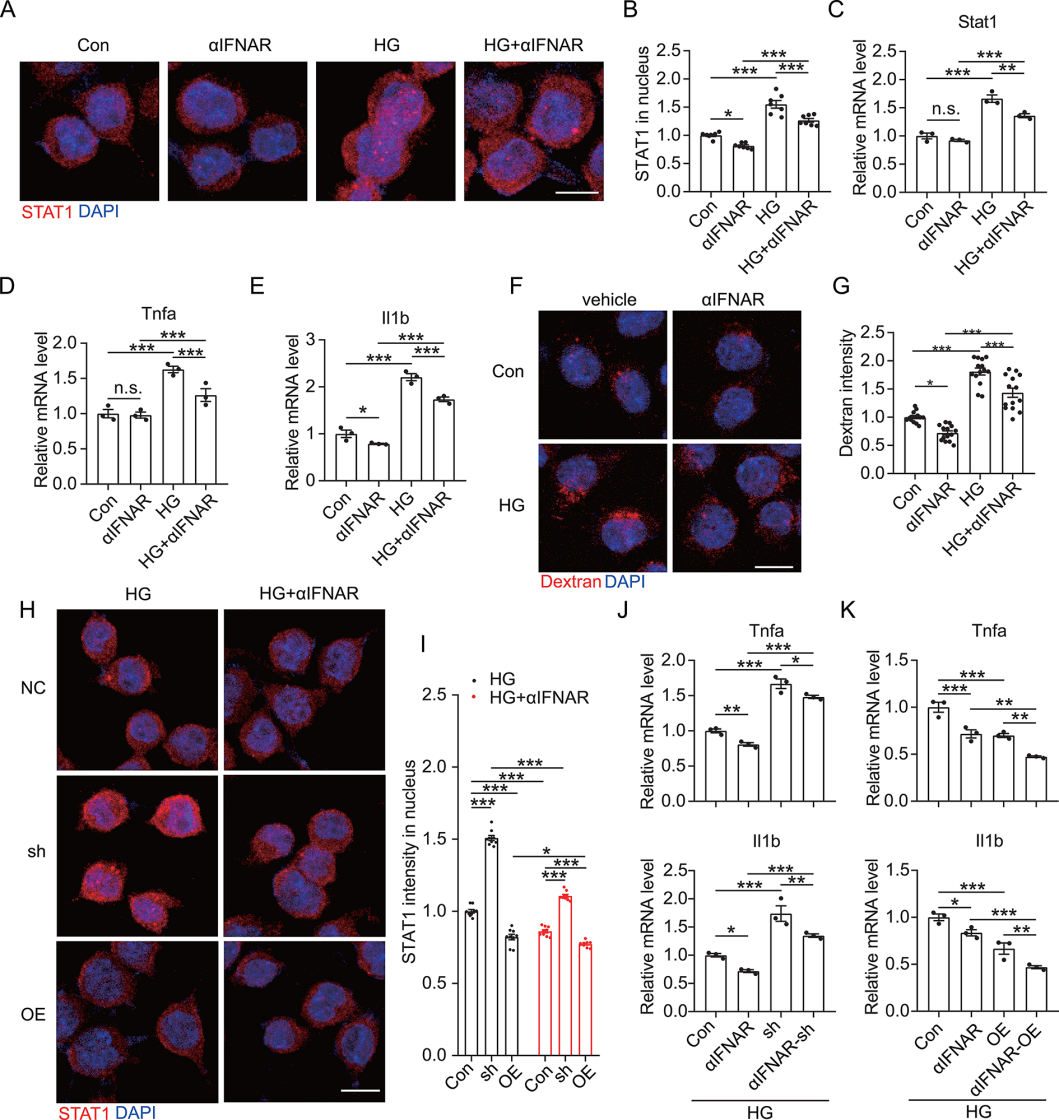
Inhibition of IFNAR1 attenuates HG-induced proinflammatory polarization of microglia. (**A**) BV2 cells were co-treated with αIFNAR1 and HG for 6 h, followed by immunofluorescence labeling of STAT1 and counterstaining with DAPI. Scale bar = 10 μm. (**B**) Nuclear STAT1 intensity in the BV2 cells in panel **A** (*n =* 7). (**C** to **E**) Expressions of *Stat1*, *Tnfa*, and *Il1b* in the BV2 cells co-treated with αIFNAR and HG were measured via qRT-PCR (*n* = 3). (**F**) BV2 cells were co-treated with αIFNAR and HG for 6 h, followed by incubation with 40-kDA TRITC-Dextran for 30 min. The cells were then fixed and stained with DAPI. Scale bar = 10 μm. (**G**) TRITC fluorescence intensity in each co-treated BV2 cell in panel **F** (*n =* 14). (**H**) shTREM2 and TREM2-OE BV2 cells were co-treated with αIFNAR and HG for 6 h, followed by labeling with anti-STAT1 antibodies and counterstaining with DAPI. Scale bar = 10 μm. (**I**) Nuclear STAT1 intensity in the shTREM2 and TREM2-OE BV2 cells in panel **H** (*n =* 9). (**J** and **K**) qRT-PCR was used to estimate *Tnfa* and *Il1b* expression in the shTrem2 BV2 cells (**J**) and TREM2-OE BV2 cells (**K**) co-treated with αIFNAR and HG (*n* = 3). *p < 0.05, **p < 0.01, and ***p < 0.001 by two-way ANOVA. n.s. indicates no significant difference

### TREM2 downregulates IFNAR1 signaling in HG-treated microglia

Further, we investigated the TREM2 mechanism involved in inhibiting the proinflammatory activation of HG-treated microglia by using RNA-seq to screen for differentially expressed genes in the microglia caused by the modulation of *Trem2*. As illustrated in Figs. 4A and 146 genes were significantly altered in the HG-treated microglia, showing a negative association with the overexpression and silencing of TREM2. Additionally, we found significant upregulation of the IFNAR-JAK-STAT pathway in the HG-treated microglia, whereas the upregulation of TREM2 reversed the upregulation of this pathway. Further KEGG analysis for the genes upregulated after HG treatment revealed that the JAK-STAT, TNF, and adipocytokine signaling pathways were all significantly activated (Fig. 4B). Furthermore, this finding suggested that TREM2 potentially regulated the activation of HG-treated microglia by modulating IFNAR signaling. Subsequent validation at the transcriptional level confirmed that HG caused the activation of IFNAR1, Tyk2, and STAT1 and that upregulated TREM2 in the HG-treated microglia significantly inhibited IFNAR1 pathway activation (Fig. 4C). Conversely, silencing TREM2 exacerbated the activation of the IFNAR1 signaling in the HG-treated microglia (Fig. 4D). Moreover, the HFD mice brains exhibited a significant upregulation of nuclear STAT1 (Fig. 4E and F), along with significantly upregulated IFNAR1, Tyk2, and STAT1 in the BLA and CA1 regions (Fig. 4G to I). These results suggested that microglial activation in the HFD mice might be related to the activation of IFNAR1 signaling, whereas the compensatory upregulation of TREM2 was potentially linked to the inhibition of IFNAR1 activation. This hypothesis was further corroborated by cellular analysis results, wherein the overexpression of TREM2 suppressed the nuclear expression of STAT1, while TREM2 interference further upregulated STAT1 nuclear expression in HG-treated microglia (Fig. s6).

**Fig. 6:**
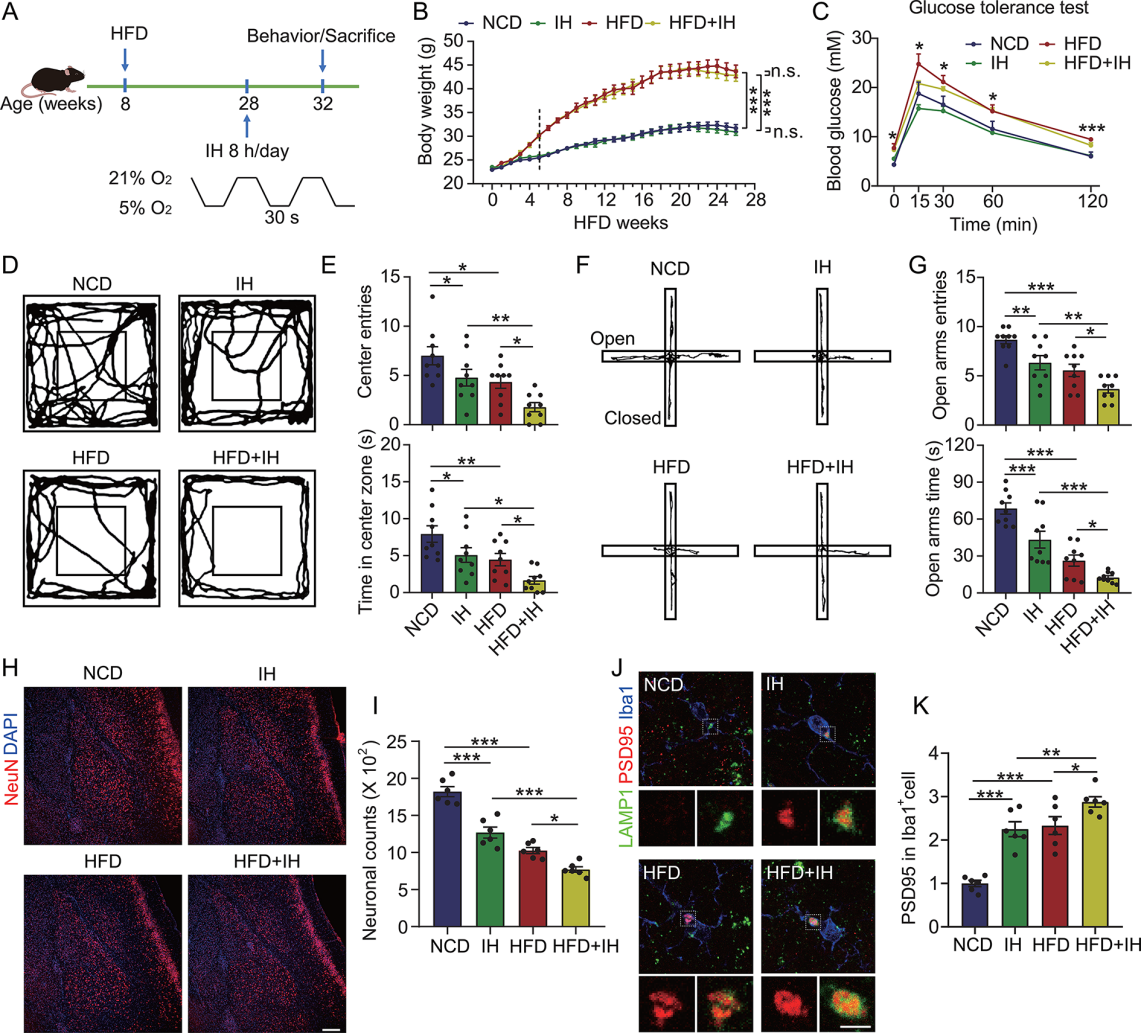
IH exacerbates anxiety-like behavior and neuronal loss in HFD-induced diabetic mice. (**A**) Flowchart of the OSA model construction. (**B**) Weekly body weights of the mice. (**C**) Glucose tolerance test results of the mice after HFD + IH treatment (*n* = 4). (**D**) Movement trajectory of the mice in the OFT. (**E**) The frequency of mice entries and their dwell times in the center zone of the open field (*n* = 9). (**F**) Movement trajectory of the mice in the EPM test. (**G**) Frequency of mice entries and the time spent by them in the open arm of the maze (*n* = 9). (**H**) Brain sections of the mice were labeled with anti-NeuN antibody and counterstained with DAPI, followed by microscopy imaging of the BLA region. Scale bar = 200 μm. (**I**) The number of neurons in the BLA region in panel **H** (*n* = 6). (**J**) Brain sections of the mice were probed with anti-LAMP1, anti-PSD95, and anti-Iba1 antibodies, followed by microscopy imaging of the microglia in the BLA region. Scale bar = 3 μm. (**K**) PSD95 intensity in the Iba1^+^ cells in panel **J** (*n* = 6). *p < 0.05, **p < 0.01, and ***p < 0.001 by two-way ANOVA

### Inhibition of IFNAR1 signaling synergizes with TREM2 to co-inhibit the proinflammatory polarization of HG-treated microglia

Next, HG-administered microglia were pretreated with αIFNAR to ascertain whether the IFNAR1 signaling pathway promoted the proinflammatory activation of HG-treated microglia. Our results showed that αIFNAR significantly inhibited the nuclear expression of STAT1 in HG-treated microglia (Fig. 5A to C), suppressed the synthesis of proinflammatory factors (Fig. 5D and E), and inhibited the synaptic phagocytosis activity of HG-treated microglia (Fig. 5F and G), suggesting that inhibiting IFNAR1 reduces the proinflammatory activation of microglia via diminished STAT1 signaling. Additionally, we verified that IFNAR1 was involved in TREM2-mediated anti-inflammatory response. Our findings demonstrated that αIFNAR significantly inhibited the nuclear expression of STAT1 in HG-treated shTrem2 microglia, with relatively greater attenuation of STAT1 nuclear expression in the HG-treated TREM2-OE microglia (Fig. 5H and I). In line with these results, αIFNAR treatment inhibited the upregulation of proinflammatory factors in HG-treated shTrem2 microglia as well as led to a further reduction in the proinflammatory factors in HG-treated TREM2-OE microglia (Fig. 5J and K). Considering these findings, the inhibition of the IFNAR1 pathway may synergize with TREM2 to jointly inhibit the proinflammatory activation of HG-treated microglia.

### IH rather than blood glucose exacerbates anxiety-like behavior and neuronal damage in HFD-induced diabetic mice

OSA, a common complication of T2DM, is known to be associated with cognitive impairment. Hence, we examined whether the OSA model constructed with IH worsened the spatial learning-memory capacity and anxiety-like behavior of the HFD-induced T2DM mice (Fig. 6A). Our findings indicated that IH treatment did not significantly alter the body weight, blood glucose (Fig. 6B and C), and spatial learning-memory capacity of the HFD mice (Fig. s7). However, IH significantly aggravated the anxiety-like behavior of these mice. In the case of the OFT, the HFD + IH mice had fewer entries and shorter dwell times in the central zone of the open field than the HFD mice (Fig. 6D and E). Similarly, the EPM results demonstrated that HFD + IH mice spent less time and made fewer entries in the open arm of the maze than the HFD mice (Fig. 6F and G). Neither distance traveled nor average speed of mice during the tests of mice changed in the OFT and EPM tests (Fig. s8). Consistent with these behavioral results, IH significantly exacerbated the loss of neurons and synapses in the BLA region (Fig. 6H and I, Fig. s9A and B); however, it had no significant effect on the synaptic number in the CA1 region (Fig. s9C and D). Additionally, the localization of PSD95 with LAMP1 was significantly greater in the Iba1^+^ cells in the BLA region of the HFD + IH mice brains than in that of the HFD mice (Fig. 6J and K). All these results suggested that IH induced neuronal loss in the BLA region and worsened anxiety-like behavior in HFD mice by specifically enhancing the synaptic phagocytosis ability of the microglia in the BLA region.

**Fig. 7:**
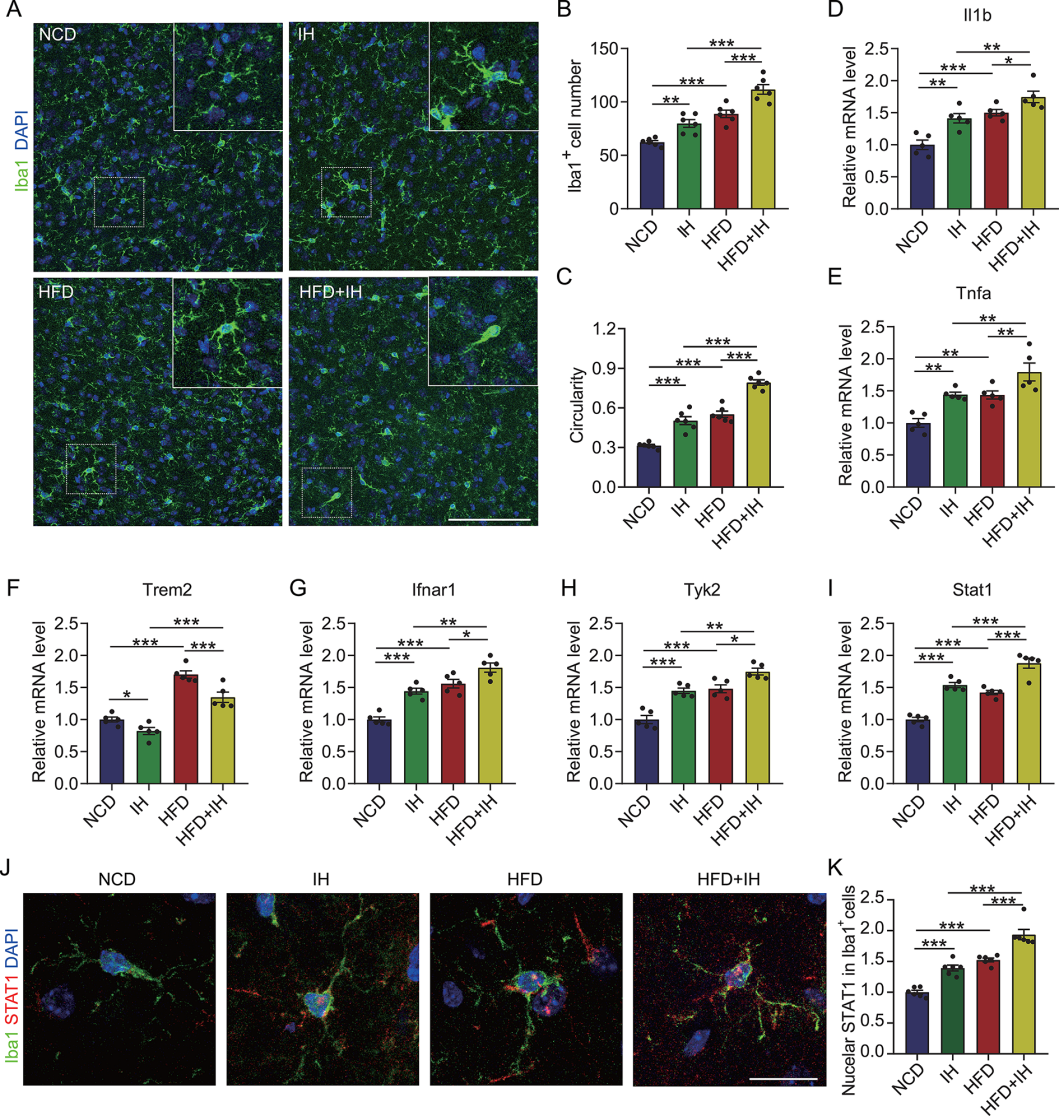
IH and HFD co-treatment promotes proinflammatory microglial polarization in the mouse brain via TREM2-IFNAR1 signaling. (**A**) Brain sections of the mice co-treated with IH and HFD were labeled with anti-Iba1 antibodies and counterstained with DAPI, followed by microscopy imaging of the BLA region. Scale bar = 100 μm. (**B**) The number of Iba1^+^ cells in panel **A** (*n* = 6). (**C**) Circularity coefficients of the Iba1^+^ cells in panel **A** (*n* = 6). (**D** to **I**) Expressions of *Il1b*, *Tnfa*, *Trem2*, *Ifnar1*, *Tyk2*, and *Stat1* in the BLA region of the brain tissue were measured using qRT-PCR (*n* = 5). (**J**) Brain sections of the mice co-treated with IH and HFD were labeled with anti-Iba1 and anti-STAT1 antibodies and counterstained with DAPI, followed by microscopy imaging of the microglia in the BLA region. Scale bar = 10 μm. (**K**) Nuclear STAT1 intensity in the Iba1^+^ cells in panel **J** (*n* = 6). *p < 0.05, **p < 0.01, and ***p < 0.001 by two-way ANOVA

**Fig. 8:**
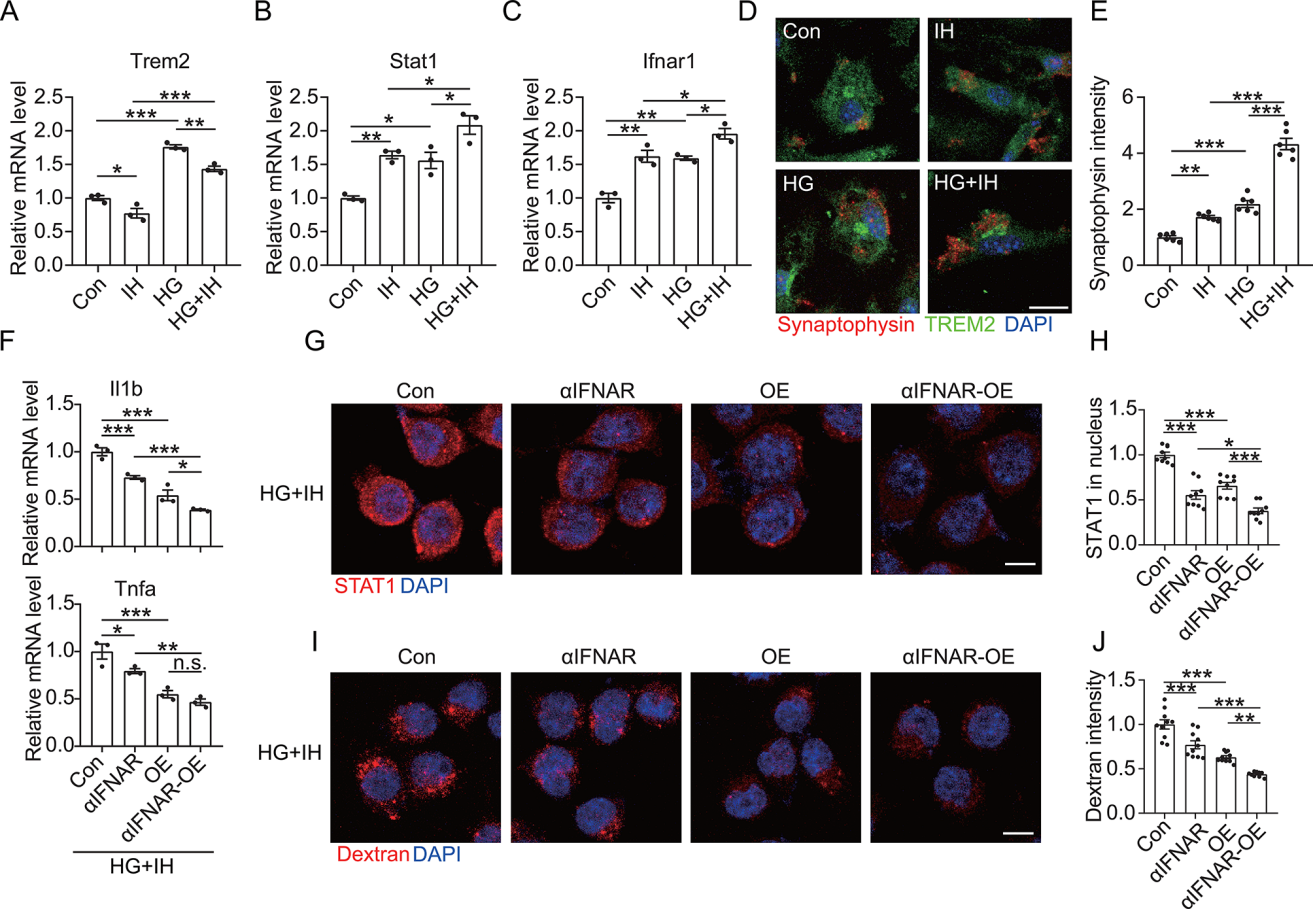
Upregulation of TREM2 inhibits proinflammatory microglial activation induced by IH and HG co-treatment. (**A** to **C**) Expressions of *Trem2*, *Stat1*, and *Ifnar1* in BV2 cells co-treated with HG and IH were detected via qRT-PCR (*n* = 3). (**D**) Primary microglia were co-treated with HG and IH and incubated with synaptosomes for 30 min. The cells were then labeled with anti-TREM2 and anti-synaptophysin antibodies and counterstained with DAPI. Scale bar = 20 μm. (**E**) Synaptophysin intensity in the TREM2^+^ cells in panel **D** (*n* = 6). (**F**) Expressions of *Il1b* and *Tnfa* in TREM2-OE BV2 cells were estimated by qRT-PCR (*n* = 3). (**G**) TREM2-OE BV2 cells were co-treated with αIFNAR, HG, and IH, followed by probing with anti-STAT1 antibodies and counterstaining with DAPI. Scale bar = 10 μm. (**H**) Nuclear STAT1 intensity per cell in panel **G** (*n* = 9). (**I**) TREM2-OE BV2 cells were treated with αIFNAR, HG, and IH and then incubated with 40-kDa TRITC-Dextran for 30 min. Finally, the cells were fixed and stained with DAPI. Scale bar = 10 μm. (**J**) TRITC intensity per cell in panel **I** (*n* = 9). *p < 0.05, **p < 0.01, and ***p < 0.001 by two-way ANOVA. n.s. indicates no significant difference

### IH increases proinflammatory microglial activation by modulating TREM2-regulated IFNAR1 signaling

Additionally, we assessed the number and morphology of brain microglia to verify whether IH aggravates neuronal damage in the BLA region of the HFD mice by increasing microglial activation. As illustrated in Fig. 7A to C, IH increased the number and the circularity coefficients of the microglia in the BLA region of the HFD mice. Furthermore, IH escalated the expression of proinflammatory factors in the HFD mice brains (Fig. 7D and E). IH also caused TREM2 downregulation in the HFD mice brains (Fig. 7F). This downregulation of TREM2 aligned with the IH-induced amplification of the IFNAR1 pathway expression (Fig. 7G to I) and increased STAT1 nuclear expression (Fig. 7J and K). Therefore, IH potentially exacerbates microglial proinflammatory activation in HFD mice by inhibiting TREM2. We also found that both IFN-α and IFN-β were upregulated in the brains of HFD mice, and IH further exacerbated the elevation of IFN-α and IFN-β (Fig. s10), which was consistent with the trend of IFNAR, indicating a complex regulatory mechanism of the IFN signaling pathway.

We further verified the effect of IH on TREM2-IFNAR1 signaling in the HG-treated microglia in vitro. As depicted in Fig. 8A to C, IH inhibited TREM2 and upregulated the IFNAR1 pathway in HG-treated microglia. IH was also found to heighten the synaptic phagocytosis activity of the HG-treated microglia (Fig. 8D and E). Conversely, both αIFNAR treatment and TREM2 overexpression reversed the proinflammatory activation (Fig. 8F), nuclear expression of STAT1 (Fig. 8G and H), and aberrant phagocytosis (Fig. 8I and J) in the HG + IH microglia. This finding indicated that TREM2 inhibition by IH leads to the activation of the IFNAR1 signaling pathway, thereby triggering the proinflammatory activation and increased synaptic phagocytosis activity of microglia.

## Discussion

Mice with T2DM induced by a 60% kcal HFD are typically characterized by weight gain, elevated fasting glucose, decreased glucose tolerance, insulin resistance, and impaired cognition [24, 44]. Hong Zhuang et al. reported that the 60% kcal HFD-induced T2DM mice exhibited an increased number of activated hippocampal microglia, altered microglial morphology, along with anxiety- and depression-like behaviors as well as memory impairments [45]. A study by Xiangyu Guo et al. simulated T2DM combined with OSA by administering IH to KK-Ay mice and revealed that IH inhibited autophagy and exacerbated apoptosis in the hippocampal neurons via the HMGB1/TLR4 pathway. Another investigation by Yu Shi et al. demonstrated that IH significantly aggravated cognitive impairment in KK-Ay mice [12]. This finding was inconsistent with our study results, where we observed that IH did not worsen cognitive deficits but significantly heightened anxiety-like behaviors in the HFD mice. Previous epidemiologic studies have also indicated that OSA significantly increases anxiety in patients with T2DM [8, 46]. Interestingly, a 14-year continuous follow-up study by Lin Sun et al. highlighted that a higher proportion of older adults with anxiety symptoms progressed to cognitive impairment (39.2%) than those without anxiety symptoms (22.6%), implying that anxiety might increase the risk of developing cognitive impairment by damaging axons or synapses [47]. Studies have also shown that anxiety increases the risk of developing dementia in patients with mild cognitive impairment [48] and may serve as a predictor of cognitive decline in those with Parkinson’s disease [49]. All these studies suggest that anxiety induces and increases the progression of cognitive impairment. Moreover, the diabetic condition of HFD-induced T2DM mice may be milder than that of KK-Ay mice; therefore, anxiety, which is more sensitive to environmental changes, is the first symptom of brain-related changes. We hypothesize that the IH-treated HFD mice will eventually display cognitive impairment as the disease progresses. Moreover, we found that the average speed and movement distance of the HFD mice did not change during the tests. In the study of Jian Han et al., HFD mice were subjected to 60 min of OFT and exhibited reduced mobility during the test [50]. We suspected that HFD mice, whose stamina are significantly compromised due to over-obesity, exhibit significant under-strength during prolonged exercise.

Our study revealed that abnormal microglial activation was closely associated with anxiety and cognitive deficits in the HFD mice. Earlier clinical studies have also observed significant microglial activation in the hypothalamic region of patients with T2DM, along with an enhancement in cell number and cytosolic area [51]. Furthermore, neuroinflammation and neurological damage due to aberrant microglial activation is a distinctive feature of cognitive impairment and anxiety in T2DM animals, wherein the proinflammatory activation of hippocampal microglia in T2DM has been suggested to exacerbate cognitive deficits and anxiety-like behaviors through neuroinflammation [42]. Conversely, inhibiting microglial activation significantly improves diabetes-associated cognitive deficits and anxiety-like behaviors [42, 52]. Microglia have also been demonstrated to induce the release of excessive inflammatory cytokines via the NF-κB pathway in AD mice, triggering neuroinflammatory responses, synaptic and neural damage, and cognitive deficits [53]. All these study findings indicate that abnormal microglial activation may have a central regulatory role in various cognitive impairments and emotional disorders associated with diseases such as T2DM, and the inhibition of proinflammatory microglial activation may be a potential therapeutic target with broad applications in the treatment of cognitive dysfunction and anxiety.

Additionally, our study highlighted that TREM2 was upregulated in T2DM mice brains, consistent with prior findings. Earlier studies have shown that TREM2 overexpression improved spatial learning and memory, upregulated synapse-related proteins to alleviate synaptic transmission and ultrastructure, and inhibited neuroinflammation in T2DM mice [24]. TREM2 has also been demonstrated to attenuate neuroinflammation and neurological damage via Akt/CREB/BDNF signaling as well as ameliorate cognitive impairment and anxiety in a mouse model of traumatic brain injury [54]. The upregulation of TREM2 in the HFD mice brains may have been a compensatory response to mitigate neurological injury. Based on this notion, the increased exacerbation of anxiety in T2DM mice with comorbid OSA may be due to the decrease in TREM2, which induces a further increase in proinflammatory microglia and additional neurological destruction.

Our study proposes a novel mechanism of TREM2 neuroprotection and identifies for the first time a key regulatory role of TREM2 in the interferon pathway. Given that IFN-I-mediated immune responses are pivotal in diabetes development, IFN-I activation not only increases susceptibility to type I diabetes [55] but also exacerbates diabetes progression by initiating and enhancing inflammatory responses [56]. Conversely, blocking IFNAR has been shown to disrupt IFN-I signaling and inhibit the onset and progression of diabetes mellitus [55, 57]. Our study findings indicated that IFNAR signaling has a regulatory role in cognitive and anxiety behaviors in T2DM, suggesting that targeting IFNAR could ameliorate neurological injury in T2DM. Other studies have also reported that the IFNAR1 pathway is involved in the development of cognitive impairment and anxiety in several neurological diseases [38, 58]. Thus, TREM2 upregulation or IFNAR1 inhibition may serve as an effective treatment for cognitive impairment in patients with various neurological or non-neurological disorders.

IFN-I is a central coordinator of the immune response with antiviral, antitumor, and immunomodulatory properties. IFN-I also mediates the pathogenesis of inflammatory autoimmune diseases and infectious diseases via the aberrant activation of inflammatory responses [59]. Based on the regulation of the IFNAR1 pathway by TREM2, we hypothesize that TREM2 modulation may serve as a therapeutic modality for autoimmune diseases. Our study represents the first investigation of the interaction between the two membrane receptors TREM2 and IFNAR1 as a potential mechanism of neuronal injury and protection. Furthermore, we highlight a critical regulatory role of TREM2 in the interferon pathway, thereby providing a new perspective on the anti-inflammatory effects of TREM2 and offering novel ideas for developing interventions for neurodegenerative diseases. Lastly, we propose a novel mechanism of anxiety exacerbation in T2DM complicated with OSA and present valuable data for improving the prevention and treatment strategies.

Microglial IFNAR1 is involved in the development of cognitive impairment and anxiety associated with multiple diseases. In AD mice, long-term blockade of microglial IFNAR1 reduces cell proliferation and inflammation, thereby attenuating synaptic damage and cognitive impairment [38]. As a downstream of IFNAR1, STAT1 is involved in regulating synaptic plasticity and cognitive function, modulating microglia polarization and inflammation. Downregulation of STAT1 attenuates atherosclerosis and cognitive impairment in T2DM patients [60]. Microglia STAT1 deficiency significantly reduces synaptic dysfunction and cognitive impairment associated with Tau accumulation in AD mice [61]. The above studies suggested an important role of microglial IFNAR1-STAT1 signaling in cognitive impairment. The present study further suggests the interaction of TREM2 and IFNAR1 signaling. Ruganzu et al. reported that overexpression of TREM2 inhibits the JAK/STAT/SOCS pathway to reduce neuroinflammation and attenuate cognitive deficits in AD mice [62], which supports our findings and underscores the potential significance of our work.

Due to the shared mechanisms among Type-1-diabetes, T2DM and AD, researchers proposed AD as “Type-3-Diabetes”. Indeed, the association between T2DM and AD is complex that both are interlinked with impaired insulin and IGF actions, oxidative stress, mitochondrial dysfunction, advanced glycation end products, lipid peroxidation, and inflammation [63]. As major symptoms of OSA, disrupted sleep architecture, intermittent hypoxia and oxidative stress, intrathoracic and hemodynamic changes, and cardiovascular comorbidities, increase the risk for AD [64]. OSA increases the risk of dementia through the mediation of amyloid-β and downstream Alzheimer’s disease pathology. Improving OSA may downregulate amyloid-β load in the brain by affecting both Aβ release and clearance [65]. TREM2 exerts anti-inflammation and neuroprotection, and TREM2^R47H^ induces aberrant activation of cerebral microglia in AD mice, exacerbating cognitive deficits [66]. Overexpression of TREM2 reduces activated microglia, Aβ deposition, synaptic and neuronal loss, neuroinflammation, and attenuates cognitive impairment [62]. Thus, the present study reveals a mechanism by which TREM2 ameliorates cognitive impairment by targeting IFN signaling, which could have significant implications for AD research and prevention.

## Conclusions

The results of the present study showed that HFD upregulated the IFNAR1-STAT1 pathway and induced proinflammatory microglia, leading to synaptic damage and causing anxiety and cognitive deficits. TREM2 upregulation is a compensatory cellular response of the brains of the T2DM mice. The upregulated TREM2 exerts a negative regulation of the IFNAR1-STAT1 pathway, which in turn attenuates neurological damage. Conversely, mice with T2DM combined with OSA may exacerbate anxiety via the downregulation of TREM2, causing heightened IFNAR1-STAT1 pathway activation and consequently increasing proinflammatory microglia.

### Electronic supplementary material

Below is the link to the electronic supplementary material.

Supplementary Material 1

Supplementary Material 2

## Data Availability

No datasets were generated or analysed during the current study.

## Abbreviations

BLA: Basolateral amygdalar
DEGs: Differentially Expressed Genes
HFD: High-fat diet
NCD: Normal control diet
HG: High-glucose
OFT: Open field test
EPM: Elevated plus maze
MWM: Morris water maze
IH: Intermittent hypoxia. αIFNAR: anti-IFNAR1 antibody
IFNAR: Interferon-α/β receptor
STAT1: Signal transducers and activators of transcription 1
TREM2: Triggering receptor expressed on myeloid cells-2
T2DM: Type 2 diabetes mellitus
OSA: Obstructive sleep apnea
