# Supplementary material for: Intermittent hypoxia exacerbates anxiety in high-fat diet-induced diabetic mice by inhibiting TREM2-regulated IFNAR1 signaling
Source: J Neuroinflammation. 2024 Jul 2;21:166. doi: 10.1186/s12974-024-03160-1 (PMC11218348; doi:10.1186/s12974-024-03160-1)
Supplement: Supplementary file 1 — Supplementary Material 1 [file 12974_2024_3160_MOESM1_ESM.docx]

**Supplementary Figures**


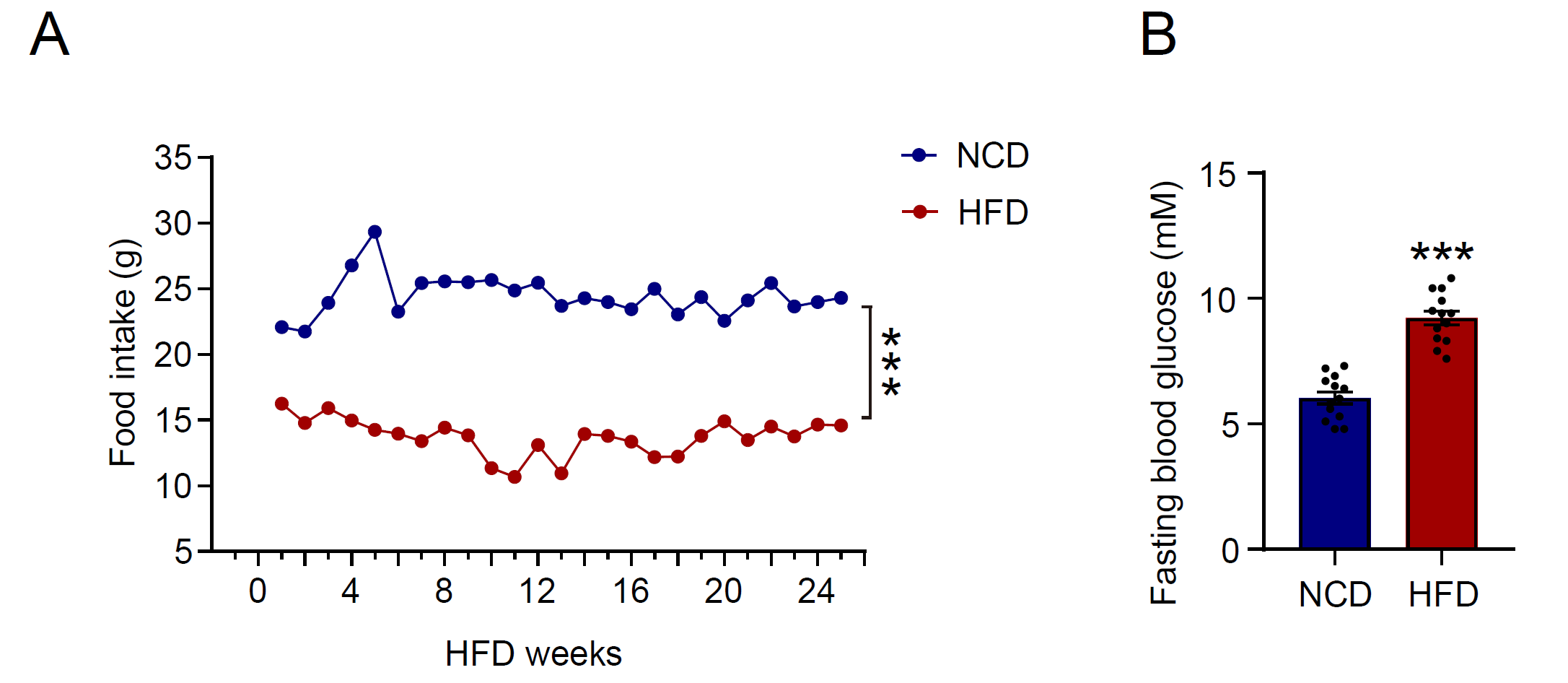


**Figure s1 HFD-induced T2DM mice demonstrate decreased food intake and elevated fasting blood glucose.** (**A**) Average weekly food intake of the mice. (**B**) Random blood glucose levels of the mice after 26 weeks of HFD. *n* = 13, ****p* < 0.001 by Student’s *t*-test.


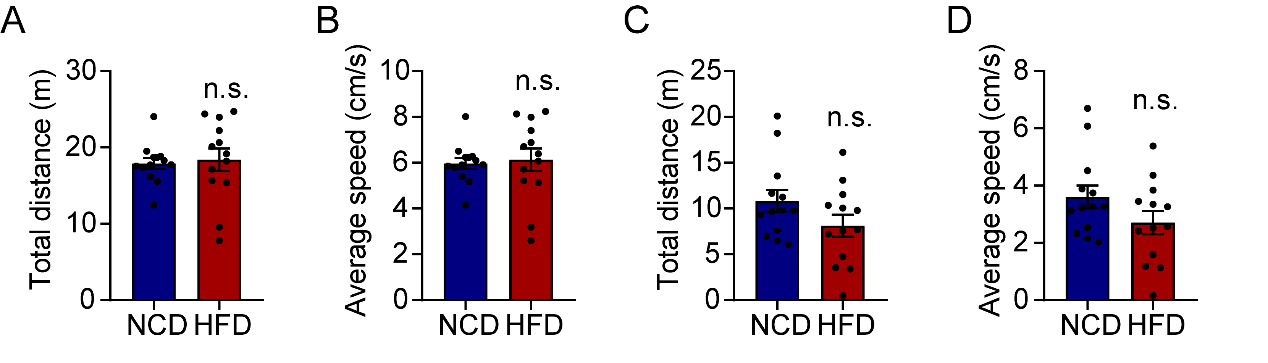


**Figure s2 HFD has no significant effect on the total distance and average speed of the mice.** (**A**) Total distance traveled by the mice during the OFT. (**B**) Average speed of the mice during the OFT. (**C**) Total distance traveled by the mice during the EPM. (**D**) Average speed of the mice during the EPM. *n* = 13. n.s. indicates no significant difference by Student’s *t*-test.


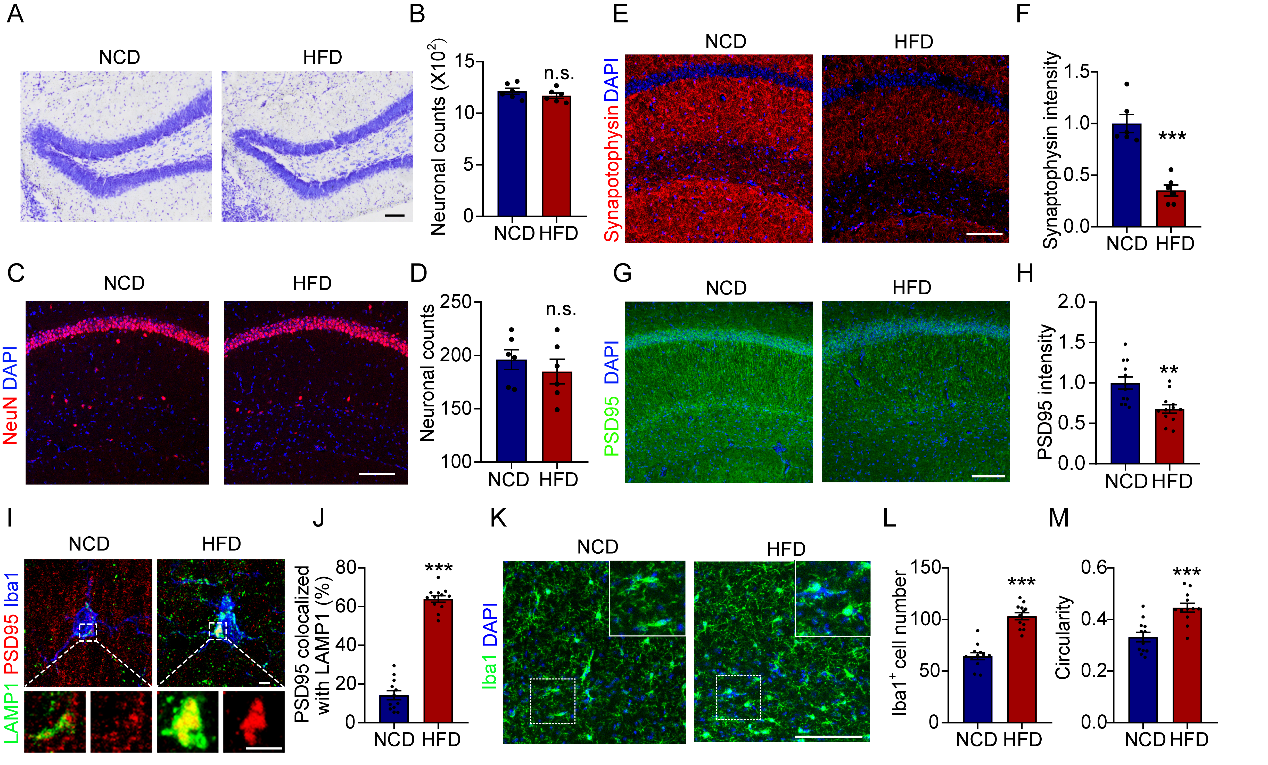


**Figure s3 HFD induces microglial activation and synaptic phagocytosis in the CA1 region of the mouse brain.** (**A**) Brain sections of the HFD mice underwent Nissl staining to identify neuronal nuclei in the microscopy images of the hippocampal DG region. Scale bar = 200 μm. (**B**) The number of neurons in the DG region in panel **A** (*n* = 6). (**C**) Brain sections of the mice were labeled with anti-NeuN antibodies followed by microscopy imaging of the CA1 region. Scale bar = 100 μm. (**D**) The number of neurons in the CA1 region in panel **C** (*n* = 6). (**E** and **G**) Brain sections of the HFD mice were labeled with anti-synaptophysin (**E**) or anti-PSD95 (**G**) antibodies, and counterstained with DAPI, followed by imaging of the CA1 region. Scale bar = 100 μm. (**F** and H) Synaptophysin (**E**) or PSD95 (**G**) intensity in the CA1 region (*n* = 6). (**I**) Brain sections of the HFD mice were labeled with anti-LAMP1, anti-PSD95, and anti-Iba1 antibodies to visualize the microglia in the CA1 region. Scale bar = 3 μm. (**J**) Colocalization ratio of PSD95 and LAMP1 in the Iba^+^ cells in panel **I** (*n* = 12). (**K**) Brain sections of the HFD mice were labeled with anti-Iba1 antibody and counterstained with DAPI to observe the CA1 region. Scale bar = 100 μm. (**L**) The number of Iba1^+^ cells in panel **K** (*n* = 12). (**M**) Circularity coefficients of the Iba1^+^ cells in panel **K** (n = 12). ***p* < 0.01, ****p* < 0.001 by Student’s *t*-test. n.s. indicates no significant difference.


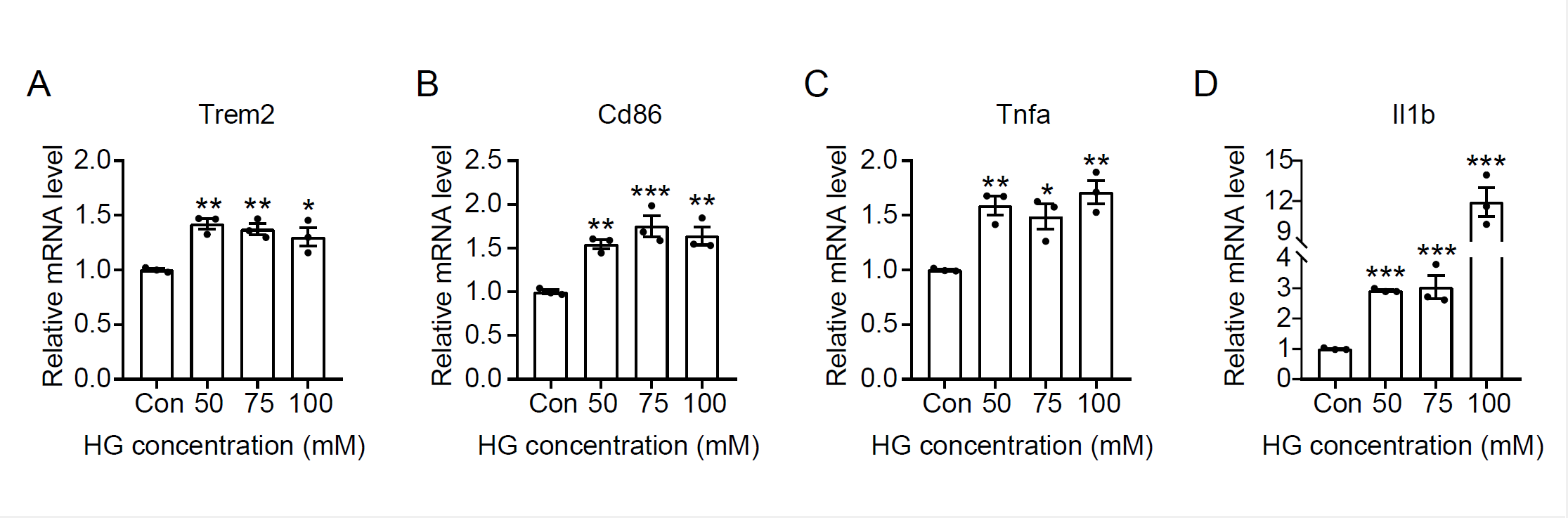


**Figure s4 HG treatment induces TREM2 upregulation and proinflammatory microglial activation.** (**A** to **D**) BV2 cells were treated with different glucose concentrations for 6 h, and the expressions of *Trem2*, *Cd86*, *Tnfa*, and *Il1b* were determined using qRT-PCR (*n* = 3). ****p* < 0.001 by one-way ANOVA.


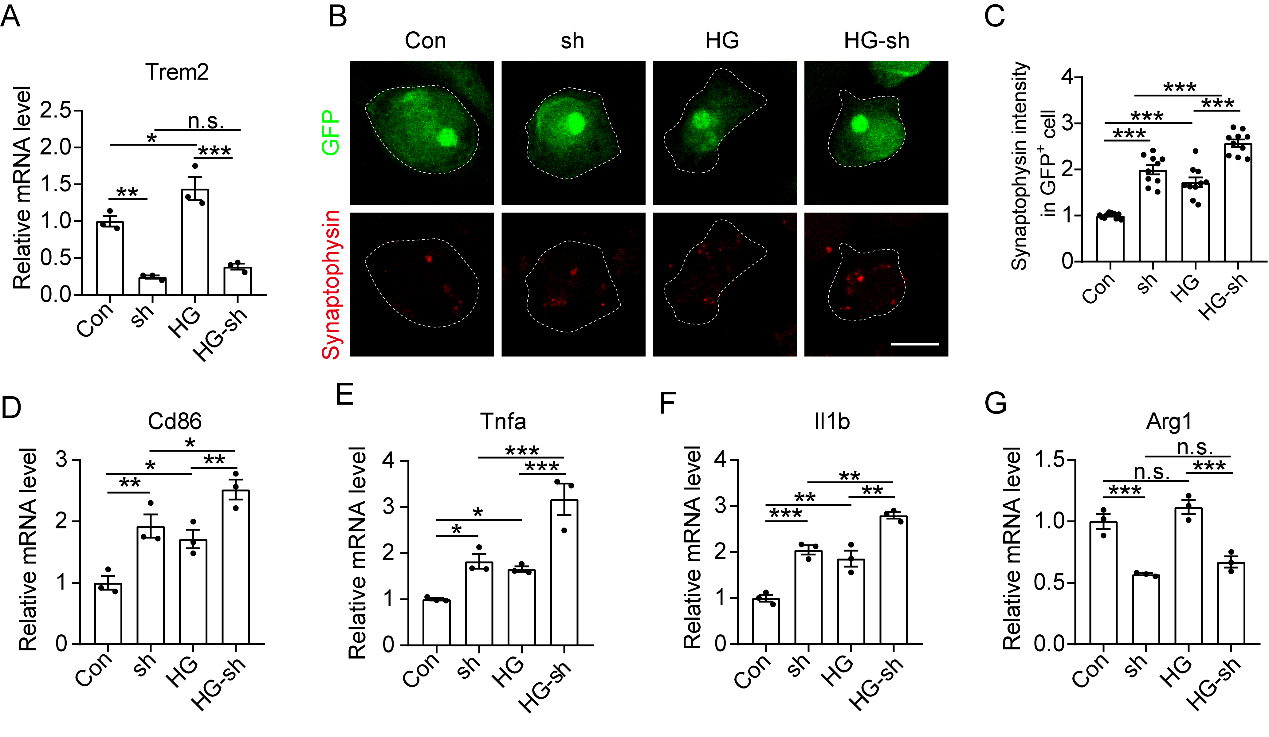


**Figure s5 Downregulation of TREM2 exacerbates the proinflammatory activation and synaptic phagocytosis activity of HG-treated microglia.** shTrem2 BV2 or primary microglia were treated with HG for 6 h. (**A**) *Trem2* expression in the BV2 cells was detected via qRT-PCR (*n* = 3). (**B**) Primary microglia were incubated with synaptosomes for 30 min, followed by immunofluorescence labeling of synaptophysin. The GFP^+^ cells were then transfected with lentivirus expressing shTrem2. Scale bar = 20 μm. (**C**) Synaptophysin intensity in the GFP^+^ cells in panel **B** (*n =* 10). (**D** to **G**) Expressions of *Cd86*, *Tnfa*, *Il1b*, and *Arg1* in BV2 cells were assessed with qRT-PCR (*n* = 3). **p* < 0.05, ***p* < 0.01, and ****p* < 0.001 by two-way ANOVA. n.s. indicates no significant difference.


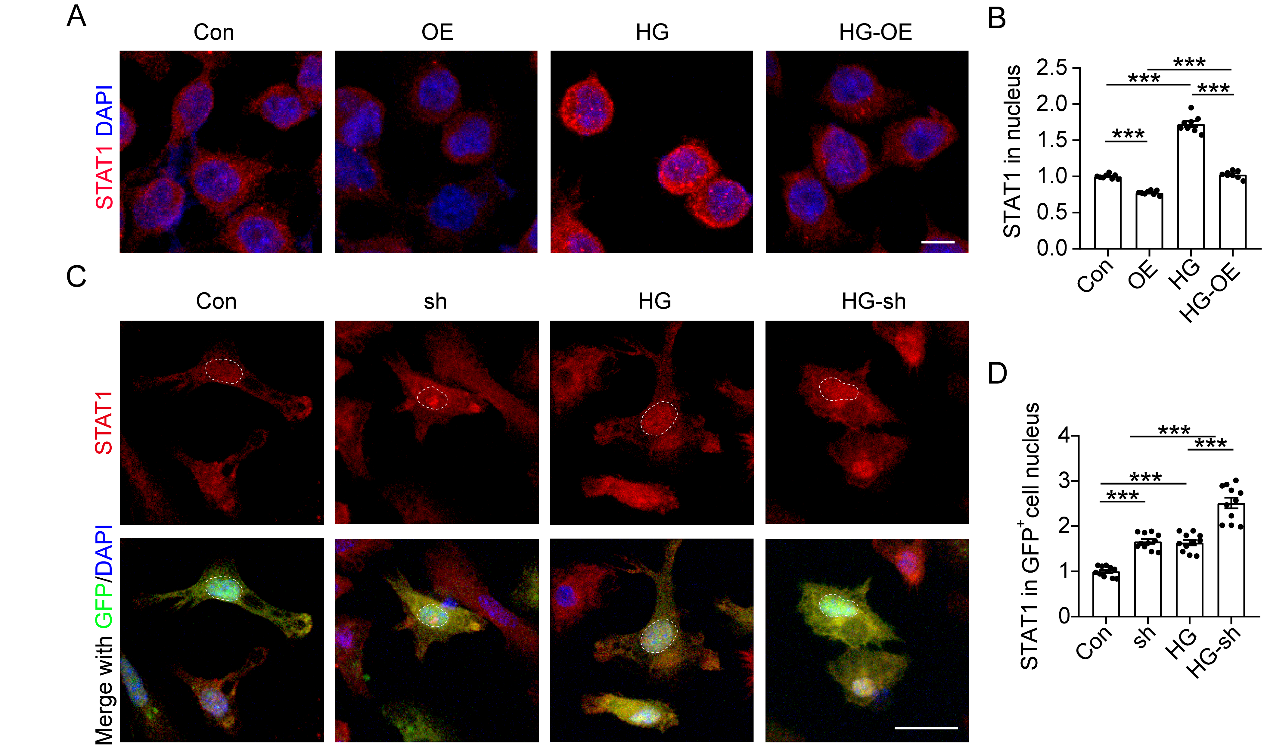


**Figure s6 TREM2 negatively regulates the nuclear expression of STAT1 in HG-treated microglia.** (**A**) TREM2-OE BV2 cells were treated with HG for 6 h, followed by labeling with anti-STAT1 antibodies and counterstaining with DAPI. Scale bar = 10 μm. (**B**) Nuclear STAT1 intensity in the TREM2-OE BV2 cells in panel **A** (*n* = 8). (**C**) Primary microglia transfected with shTrem2-expressing lentivirus (GFP^+^) underwent HG treatment for 6 h. The cells were then labeled with anti-STAT1 antibodies and counterstained with DAPI. Scale bar = 20 μm. (**D**) Nuclear STAT1 intensity in the GFP^+^ cells in panel **C** (*n* = 11). ****p* < 0.001 by two-way ANOVA.


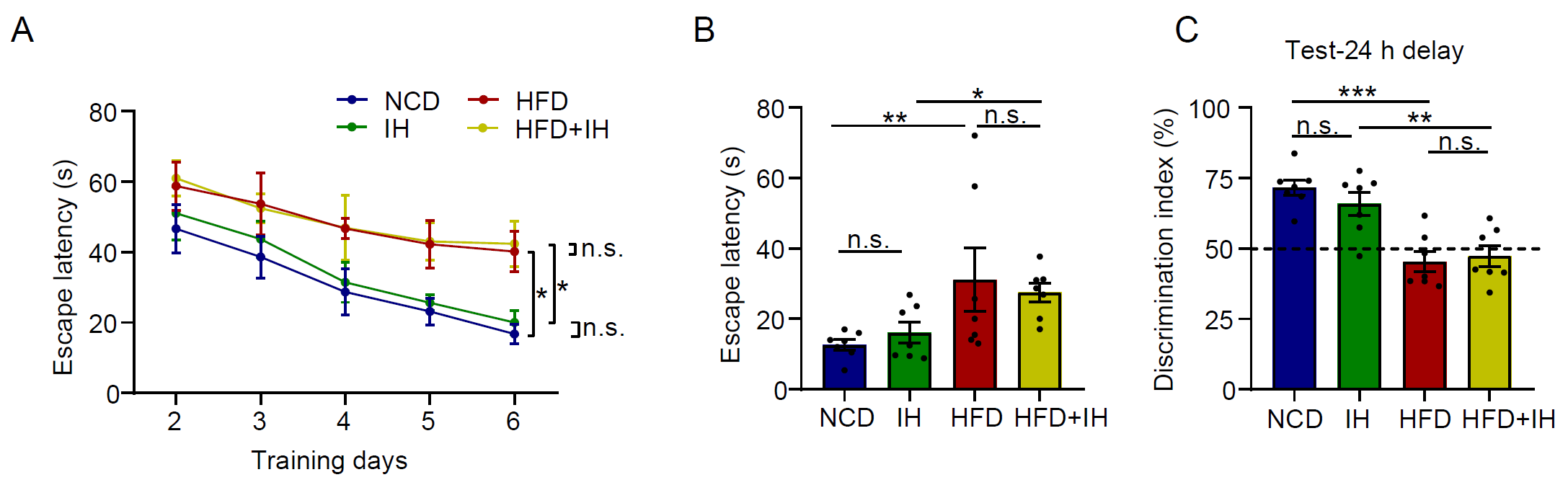


**Figure s7 IH has no significant effect on the cognition of HFD mice.** (**A**) Escape latency (i.e., time to reach the target platform) of the mice during the training period of the MWM test. (**B**) Escape latency of the mice to reach the target for the first time during the probe period of the MWM test. (**C**) Discrimination index of the mice for the novel object in the NOR test after a delay of 24 h. n = 7. **p* < 0.05, ***p* < 0.01, and ****p* < 0.001 by two-way ANOVA. n.s. indicates no significant difference.


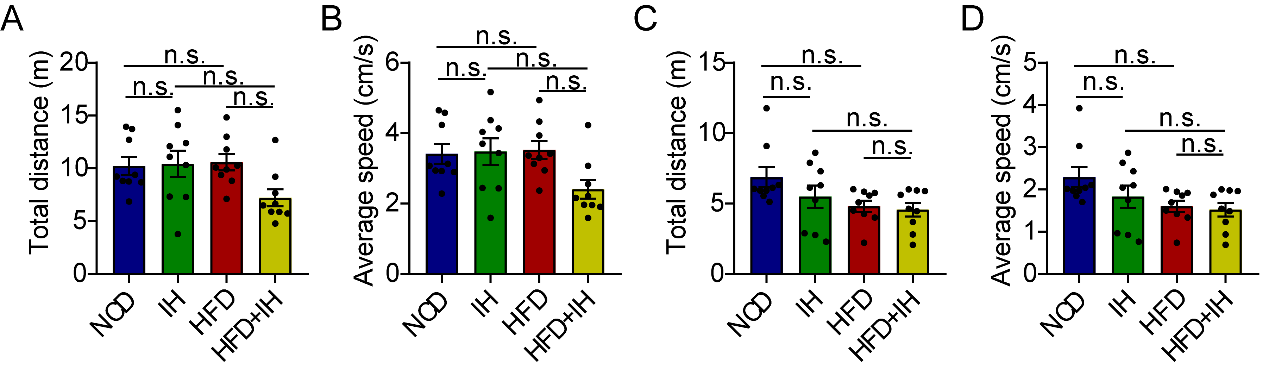
 **Figure s8 IH has no significant effect on the locomotion of the mice.** (**A**) Total distance traveled by the mice during the OFT. (**B**) Average speed of the mice during the OFT. (**C**) Total distance traveled by the mice during the EPM. (**D**) Average speed of the mice during the EPM. *n* = 9. n.s. indicates no significant difference by two-way ANOVA.


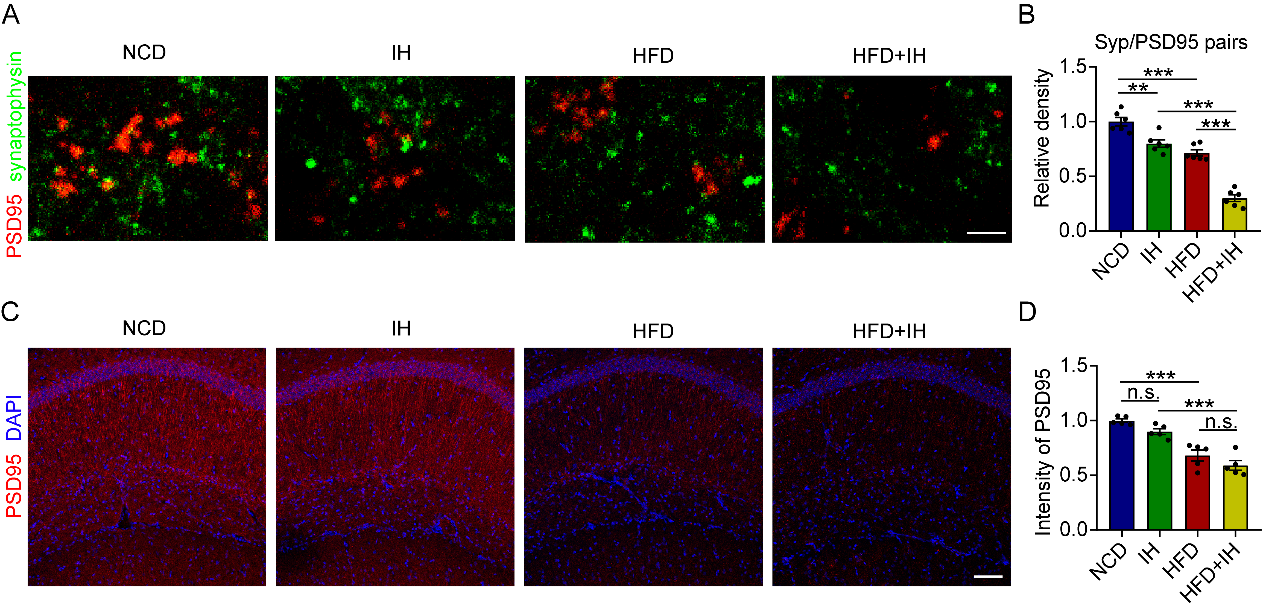


**Figure s9 IH aggravates synaptic loss in BLA region rather than in CA1 region of HFD mice**. (**A**) Brain sections of the mice were labeled with anti-PSD95 and anti-synaptophysin antibodies to acquire microscopy images of the hippocampal BLA region. Scale bar = 2 μm. (**B**) Relative density of functional synapse pairs (≤ 200 nm between puncta) in panel **A**. *n* = 6. (**C**) Brain sections of the mice were labeled with anti-PSD95 antibodies and counterstained with DAPI for imaging the CA1 region. Scale bar = 100 μm. (**D**) PSD95 intensity in the CA1 region in panel **C**. *n* = 5. ****p* < 0.001 by two-way ANOVA. n.s. indicates no significant difference.


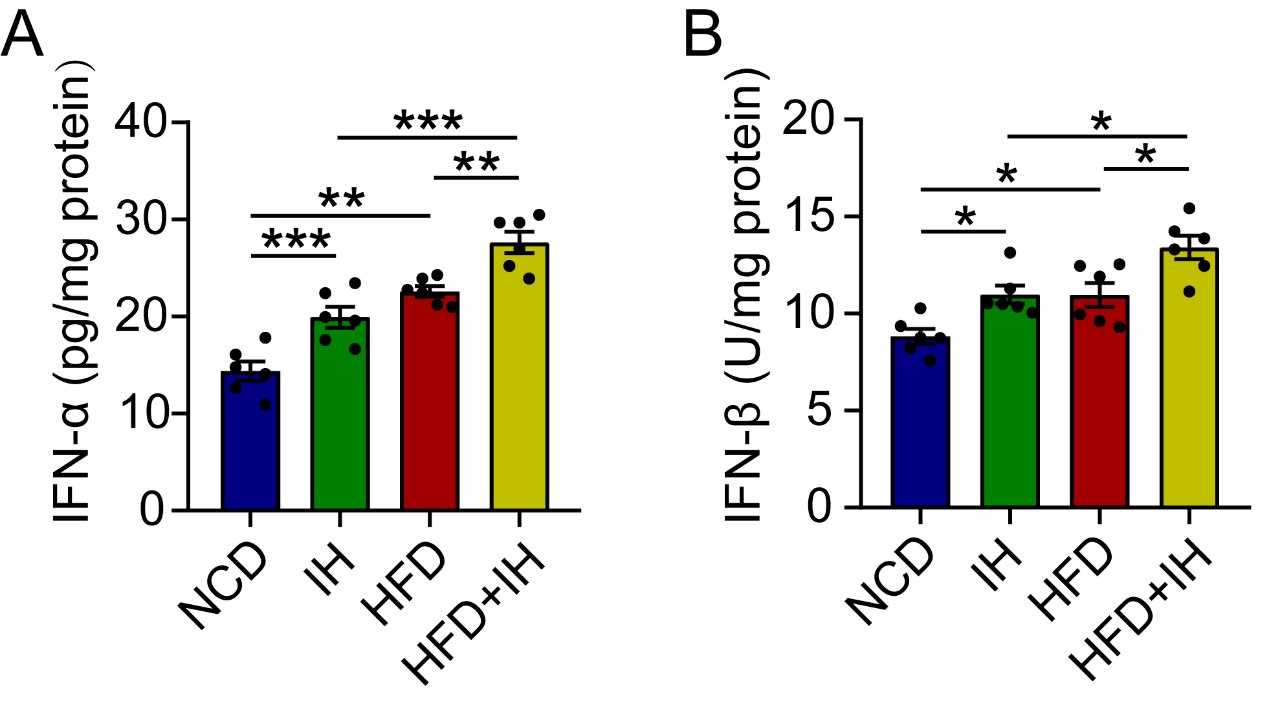


**Figure s10 IH upregulates IFN-α and IFN-β in HFD mice.** Brain tissue of HFD+IH mice were homogenized in PBS containing protease inhibitors. IFN-α (**A**) and IFN-β (**B**) Content in tissue homogenate supernatants were determined by ELISA and scaled according to protein concentration. *n* = 6. **p* < 0.05, ***p* < 0.01, and ****p* < 0.001 by two-way ANOVA.
