# Supplementary material for: Intermittent hypoxia exacerbates anxiety in high-fat diet-induced diabetic mice by inhibiting TREM2-regulated IFNAR1 signaling
Source: J Neuroinflammation. 2024 Jul 2;21:166. doi: 10.1186/s12974-024-03160-1 (PMC11218348; doi:10.1186/s12974-024-03160-1)

**Original bands**

**Antibody validation**

anti-PSD95 95 kDa


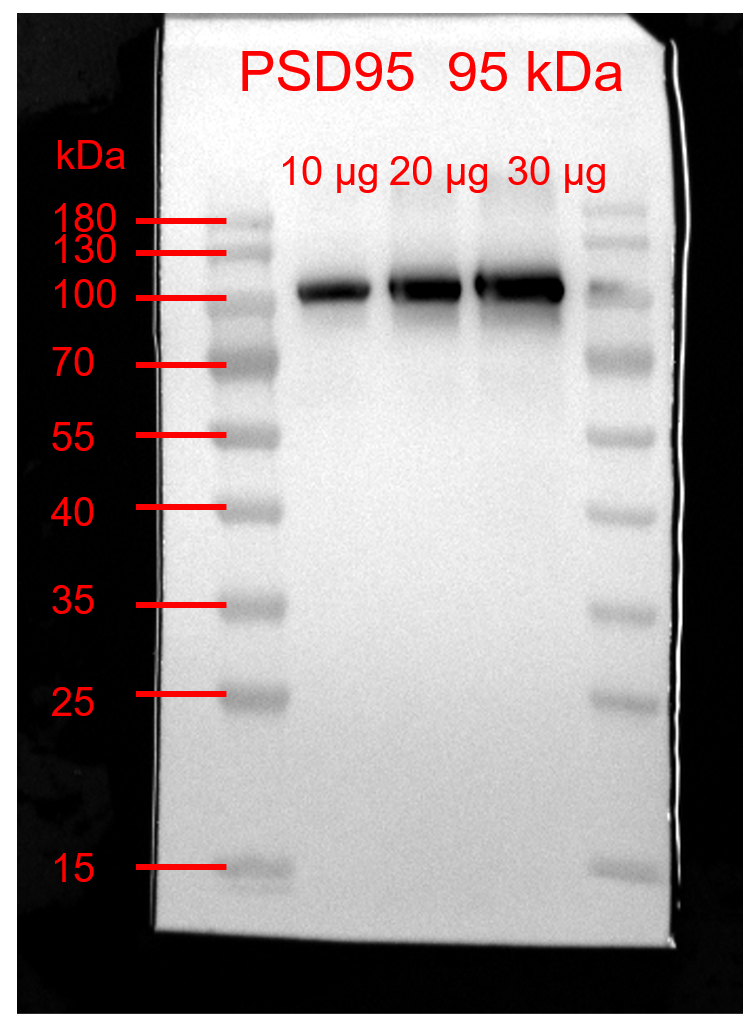


Anti-TREM2 38 kDa


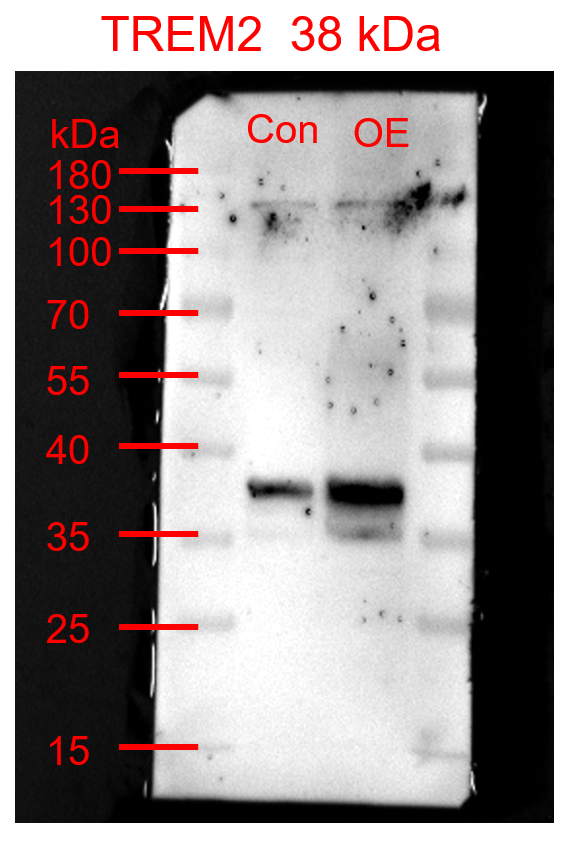

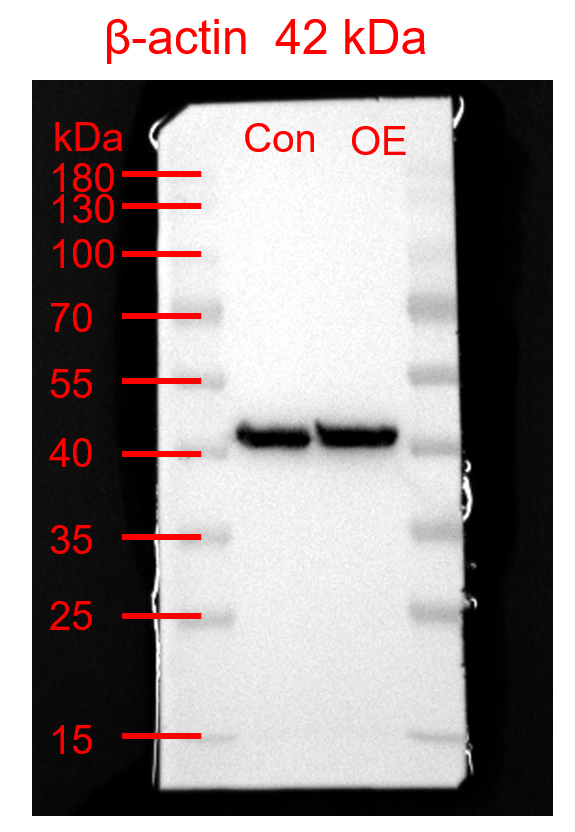


**Uncropped WB data in Figures**

**Figure 2C**


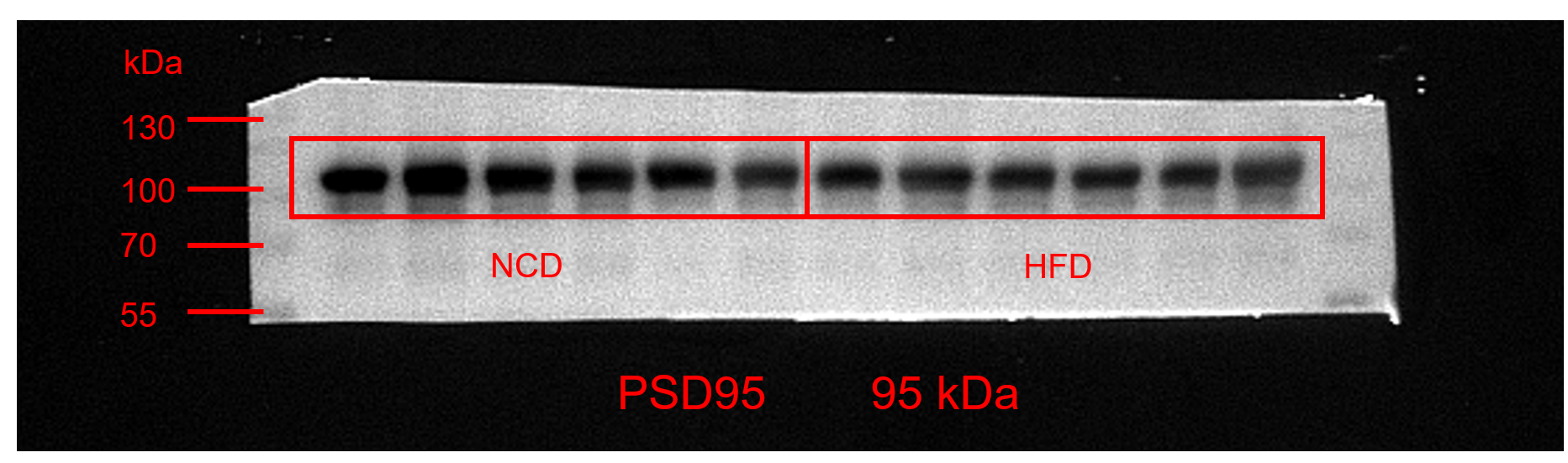


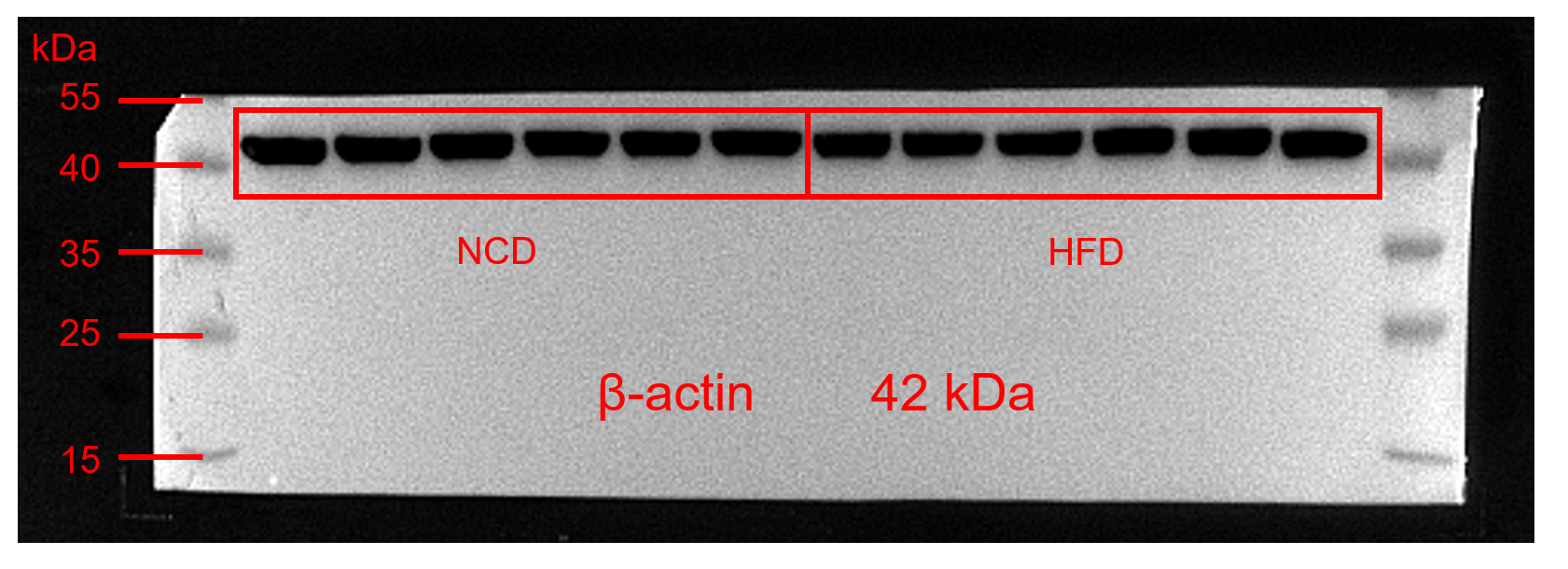


**Figure 3A**


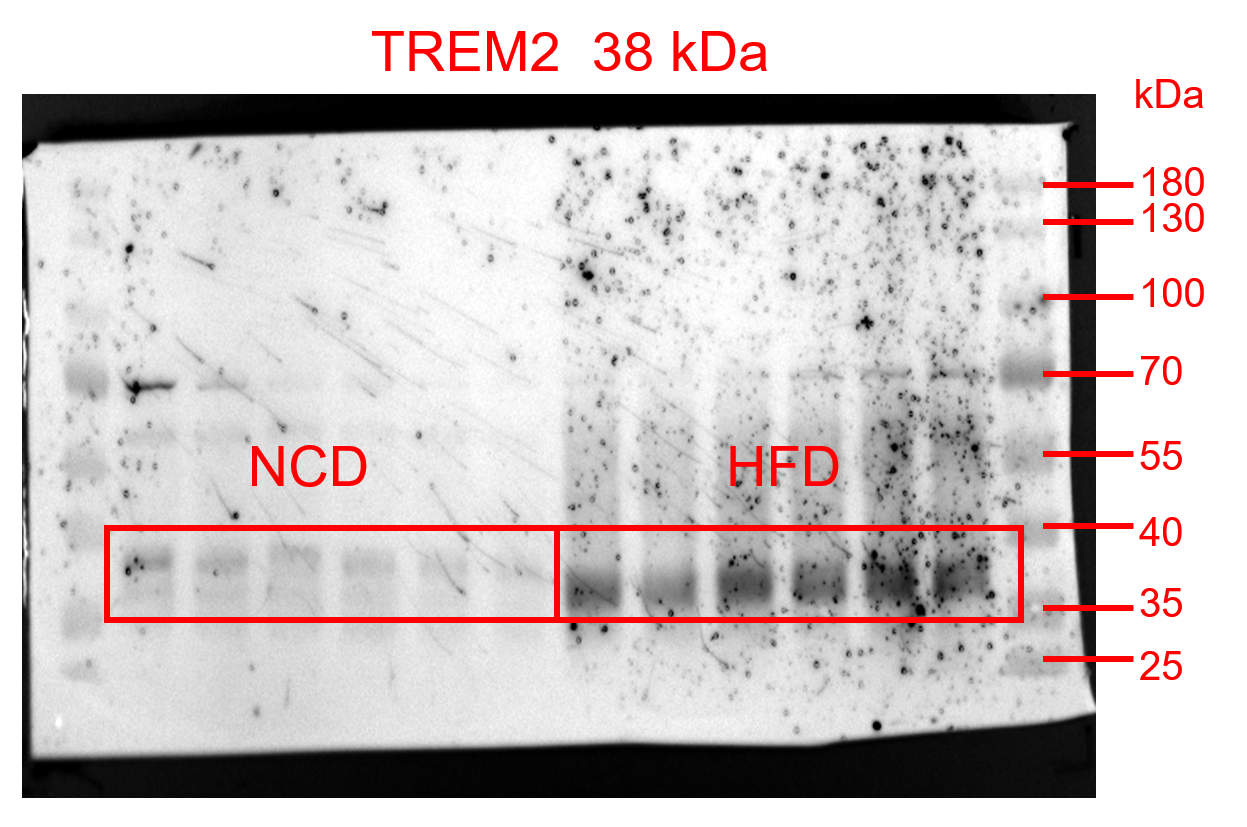


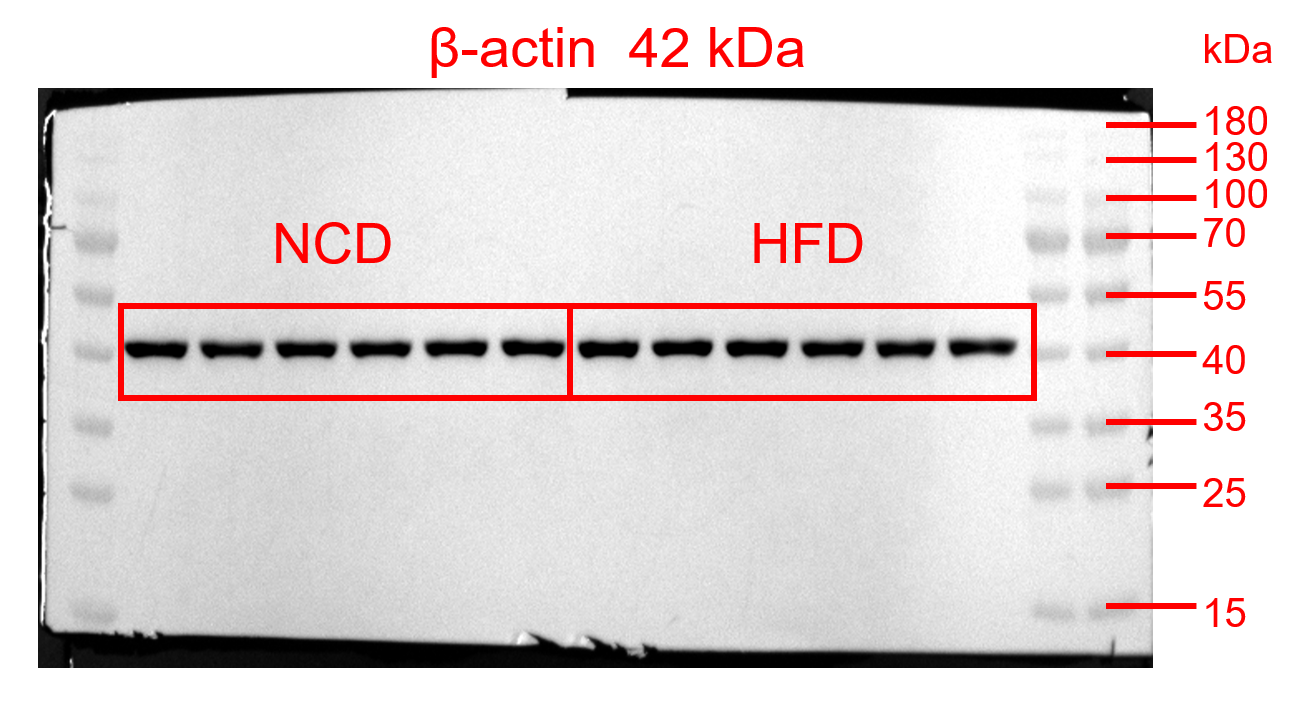

Supplement: Supplementary file 2 — Supplementary Material 2 [file 12974_2024_3160_MOESM2_ESM.docx]
